# *Drosophila* Solute Carrier 5A5 Regulates Systemic Glucose Homeostasis by Mediating Glucose Absorption in the Midgut

**DOI:** 10.3390/ijms222212424

**Published:** 2021-11-17

**Authors:** Yue Li, Weidong Wang, Hui-Ying Lim

**Affiliations:** 1 Department of Physiology, University of Oklahoma Health Sciences Center, Oklahoma City, OK 73104, USA; liyue9104@zju.edu.cn; 2 Department of Medicine, Section of Endocrinology, University of Oklahoma Health Sciences Center, Oklahoma City, OK 73104, USA; weidong-wang@ouhsc.edu

**Keywords:** solute carrier, SGLT1, glucose transporter, glucose metabolism, intestinal absorption

## Abstract

The small intestine is the initial site of glucose absorption and thus represents the first of a continuum of events that modulate normal systemic glucose homeostasis. A better understanding of the regulation of intestinal glucose transporters is therefore pertinent to our efforts in curbing metabolic disorders. Using molecular genetic approaches, we investigated the role of *Drosophila* Solute Carrier 5A5 (dSLC5A5) in regulating glucose homeostasis by mediating glucose uptake in the fly midgut. By genetically knocking down *dSLC5A5* in flies, we found that systemic and circulating glucose and trehalose levels are significantly decreased, which correlates with an attenuation in glucose uptake in the enterocytes. Reciprocally, overexpression of dSLC5A5 significantly increases systemic and circulating glucose and trehalose levels and promotes glucose uptake in the enterocytes. We showed that dSLC5A5 undergoes apical endocytosis in a dynamin-dependent manner, which is essential for glucose uptake in the enterocytes. Furthermore, we showed that the dSLC5A5 level in the midgut is upregulated by glucose and that dSLC5A5 critically directs systemic glucose homeostasis on a high-sugar diet. Together, our studies have uncovered the first *Drosophila* glucose transporter in the midgut and revealed new mechanisms that regulate glucose transporter levels and activity in the enterocyte apical membrane.

## 1. Introduction

The maintenance of normal systemic glucose homeostasis is essential for organismal physiology. Upon its absorption from the human intestinal lumen into the enterocyte, glucose travels transcellularly from the apical to the basal side of the cell and is secreted into the bloodstream to be transported to other organs [1].

Glucose absorption in the human small intestine is mediated by the sodium (Na^+^)-glucose cotransporter 1 (SGLT1), which is enriched in the apical or brush border membrane of the mature enterocytes [2]. SGLT1 is encoded by *SLC5A1*, the first of 11 gene members of the human solute carrier 5 (SLC5) family of plasma-membrane-Na^+^-dependent cotransporters that mediate the absorption of various solutes, including sugars, inositol, vitamin, iodide, and choline [3,4].

The capacity for glucose absorption in the small intestine is highly dependent on the availability of SGLT1, highlighting the importance of understanding the physiological regulation of the intestinal glucose transporter. This far, however, the mechanisms known to regulate SGLT1 are mainly elucidated by in vitro studies. Experiments performed in cultured cells and the *Xenopus* oocytes have shown that SGLT1-containing vesicles undergo exocytosis from the trans-Golgi network to the plasma membrane, where SGLT1 is incorporated [5,6,7]. Glucose regulates SGLT1 exocytosis, as shown by the observations that cellular injection of glucose or nonmetabolized glucose analogs accelerates the exocytosis of SGLT1 expressed in the *Xenopus* oocytes [5,6,7]. The incorporation of SGLT1 in the plasma membrane is balanced by the endocytosis of SGLT1 from the plasma membrane. SGLT1 has been documented to be endocytosed from the plasma membrane of cultured cells in a dynamin-independent manner [8]. Moreover, following endocytosis, SGLT1 could be targeted to the lysosomes for degradation or recycled back to the plasma membrane [8]. While these results have provided evidence supporting a role of endocytosis and downstream lysosomal degradation and recycling in regulating SGLT1 availability in the plasma membrane, they were obtained from in vitro cultured cell studies, which might not reflect the physiological role of these mechanisms in the homeostasis of glucose transporters in the enterocyte apical membrane, either under basal condition or in response to glucose stimulation. Furthermore, the endogenous function of dynamin in the apical endocytosis of glucose transporters in the enterocyte has remained unclear. Importantly, the role of endocytosis in glucose transporter-mediated glucose uptake in the enterocytes is unknown.

The *Drosophila* midgut is the functional equivalent of the mammalian small intestine and could serve as an efficient in vivo model system to study the regulation of intestinal glucose transporters. In insects, glucose is the main fuel for energy metabolism but is normally present in low concentrations in the hemolymph (insect blood) because of its reducing and osmotic properties [9]. Glucose, once absorbed from the midgut (insect equivalence of the mammalian small intestine [10]) and into the hemolymph, is converted to trehalose in the fat body [11]. Trehalose, a disaccharide consisting of two molecules of glucose, is the dominant circulating sugar in *Drosophila* and represents a permanent source of glucose that can be continuously released by enzymatic hydrolysis mediated by tissue-associated trehalases [12]. While sugars are critical substrates for insect metabolism, little is known about the transporters that mediate glucose absorption in the midgut. In *Drosophila*, up to 78 *Drosophila* genes have been found to harbor a sugar transporter motif [13]. Therefore, it is likely that a SGLT1-like glucose transporter exists in the midgut that directs dietary glucose uptake. The identification and characterization of SGLT1-like glucose transporters in the *Drosophila* midgut will likely provide new insights into human SGLT1 regulation and function. However, such glucose transporters have not yet been reported in the *Drosophila* midgut.

The *Drosophila* SLC5A (dSLC5A) family is homologous to the human SLC5 family of Na^+^-dependent solute transporters [14]. Several members of the dSLC5A family have been characterized, including dSLC5A7, which is involved in salt tolerance [15]; dSLC5A11/cupcake, which regulates feeding behavior [16]; dSLC5A15, which serves as a choline transporter [17]; and dSLC5A5, which has been implicated to be a biotin transporter/sodium-dependent multivitamin transporter (smvt) as its deficiency was associated with impaired cellular biotin uptake [18]. While various roles of the dSLC5A family members are emerging, it is still not known whether any of the dSLC5A family members functions as a glucose transporter in the midgut. Our study here is the first to show that a dSLC5A family member, dSLC5A5, exhibits properties that are consistent with a dietary glucose transporter in the *Drosophila* midgut. We further reveal previously unrecognized mechanisms that govern the abundance and activity of dSLC5A5/SGLT1 in the enterocyte apical membranes.

## 2. Results

### 2.1. Similarities between Human SGLT1 and dSLC5A5

Using the HUGO Gene Nomenclature Committee (HGNC) Comparison of Orthology *Predictions* *(HCOP)* search tool, we identified 10 genes in the dSLC5A family that are putative orthologs of human *SLC5A1* (encoding the human SGLT1/hSGLT1 glucose transporter) (available online: https://www.genenames.org/data/gene-symbol-report/#!/hgnc_id/11036 (accessed on 17 September 2021)). Among these, four dSLC5A members (dSLC5A5, dSLC5A8, dSLC5A10, and dSLC5A13) share the highest protein sequence homology with hSGLT1 (over 40% similarity and 20% identity). As it has been suggested that dSLC5A5 could be expressed in the midgut enterocytes [19], we further assessed the expression of dSLC5A5 in various tissues of the wild-type (*w^1118^*) flies. By quantitative RT-PCR (qRT-PCR), we found that *dSLC5A5* is most highly expressed in the midgut amongst other tissues, including the skeletal muscle, fat body (functional equivalence of liver and adipose tissue), and brain (Figure S1A).

To further determine the similarities between dSLC5A5 and hSGLT1, we aligned the dSLC5A5 and hSGLT1 amino acid sequences and compared them using UniProt, InterPro, and ExPASy. Overall, our analyses showed that the Na^+^-solute symporter family (SSF) signatures (underlined, Figure S2A) and all the Na^+^-binding sites (red, Figure S2A) are conserved between dSLC5A5 and hSGLT1. Moreover, our modeling by Protter revealed a similar secondary structure between hSGLT1 and dSLC5A5, with 14 transmembrane α-helices for hSGLT1 (Figures S2A (yellow highlights) and S2B) and 13 transmembrane α-helices for dSLC5A5 (Figures S2A (yellow highlights) and S2B′). Both hSGLT1 and dSLC5A5 N-termini face the intestinal lumen, while their C-termini face the cellular cytoplasm (Figure S2B,B′). Together, these results point toward several strong similarities between dSLC5A5 and hSGLT1.

### 2.2. Whole-Body Inhibition of dSLC5A5 Perturbs Systemic Glucose Homeostasis

**Figure 1:**
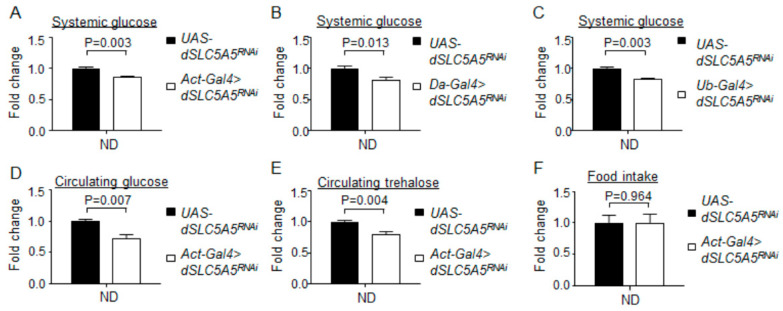
Whole-body knockdown of *dSLC5A5* perturbs systemic glucose metabolism without affecting food intake. (**A**–**D**) Systemic glucose levels in control flies (*UAS-dSLC5A5^RNAi^*) or in flies bearing the whole-body KD of *dSLC5A5* using different ubiquitous drivers, *Act-Gal4* (**A**), *Da-Gal4* (**B**), or *Ubi-Gal4* (**C**). Systemic glucose levels (μg/μL) were normalized to whole-body protein (μg/μL). (**D**,**E**) Circulating levels of glucose (**D**) and trehalose (**E**) in control flies or in flies with the whole-body KD of *dSLC5A5* mediated by *Act-Gal4* (*Act-Gal4 > dSLC5A5^RNAi^*). (**F**) Food consumption in control flies or in flies with the whole-body KD of *dSLC5A5* mediated by *Act-Gal4* (*Act-Gal4 > dSLC5A5^RNAi^*). In all cases, control flies are the RNAi transgene control flies (*UAS-dSLC5A5^RNAi^*). Results are the mean ± standard error of the mean (SEM) of 30–40 flies analyzed over at least five independent experiments and expressed as the fold change compared with that of the control flies (set at 1.0). Student’s *t*-test was used to derive *p*-values between the transgene control and KD flies. ND, normal diet.

We next examined the role of dSLC5A5 in the regulation of systemic glucose homeostasis, a process intimately associated with glucose uptake in the midgut. We found that the knockdown of *dSLC5A5* throughout the body using the ubiquitous *Gal4* drivers, *Actin-Gal4* (*Act-Gal4 > dSLC5A5^RNAi^*), *Daughterless-Gal4* (*Da-Gal4 >* *dSLC5A5^RNAi^*), or *Ubiquitin-Gal4* (*Ub-Gal4 > dSLC5A5^RNAi^*), significantly decreased whole-body glucose levels on a normal diet (ND) (Figure 1A–C). We further found significantly decreased circulating levels of glucose and trehalose in the *Act-Gal4 >* *dSLC5A5^RNAi^* flies relative to those in the control flies on an ND (Figure 1D,E). Food intake between the *Act-Gal4 >* *dSLC5A5^RNAi^* and control flies was comparable (Figure 1F), suggesting that appetite does not contribute to the decreased systemic sugar levels associated with *dSLC5A5* silencing. In all, our results suggest an important role of dSLC5A5 in the regulation of normal glucose homeostasis.

### 2.3. dSLC5A5 Functions in the Midgut to Regulate Systemic Glucose Homeostasis

As dSLC5A5 is highly expressed in the midgut (Figure S1A), we hypothesized that dSLC5A5 acts in the midgut to regulate systemic glucose homeostasis. To address this, we knocked down *dSLC5A5* with two different midgut-specific drivers, *MyoIA-Gal4* (*MyoIA-Gal4* > *dSLC5A5^RNAi^*) and *Caudal-Gal4* (*Cad-Gal4* > *dSLC5A5^RNAi^*), which resulted in the depletion of ~90% or more of the *dSLC5A5* level in the midgut (Figure S3A,B).

**Figure 2:**
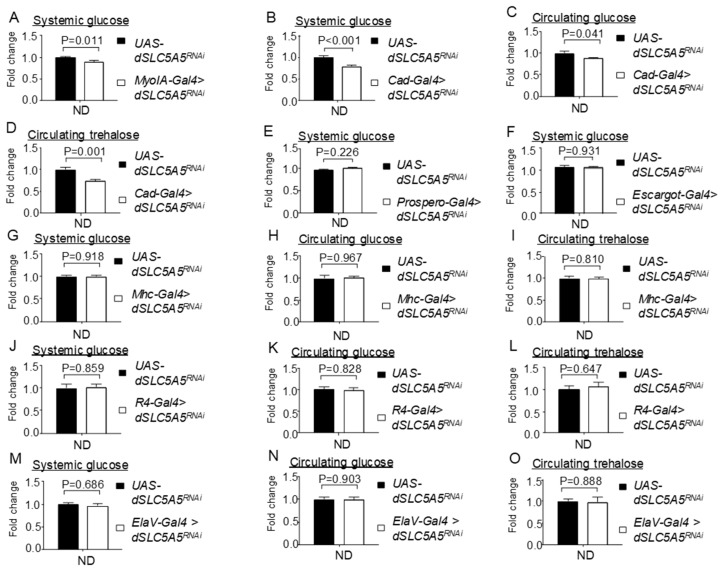
Tissue-specific knockdown of *dSLC5A5* differentially perturbs systemic glucose metabolism. (**A**,**B**) Systemic glucose levels in control flies (*UAS-dSLC5A5^RNAi^*) or in midgut-specific *dSLC5A5*-KD flies mediated by *MyoIA-Gal4* (**A**) or *Cad-Gal4* (**B**). (**C**,**D**) Circulating glucose levels (**C**) or circulating trehalose levels (**D**) in control flies or in midgut-specific *dSLC5A5*-KD flies mediated by *Cad-Gal4*. (**E**) Systemic glucose levels in control flies or in enteroendocrine cell-specific *dSLC5A5*-KD flies mediated by *Prospero-Gal4*. (**F**) Systemic glucose levels in control flies or in intestinal stem cell/enteroblast-specific *dSLC5A5*-KD flies mediated by *Escargot-Gal4*. (**G**–**I**) Systemic glucose levels (**G**), circulating glucose levels (**H**), or circulating trehalose levels (**I**) in control flies or in skeletal-muscle-specific *dSLC5A5*-KD flies mediated by *Mhc-Gal4*. (**J**–**L**) Systemic glucose levels (**J**), circulating glucose levels (**K**), or circulating trehalose levels (**L**) in control flies or in fat-body-specific *dSLC5A5*-KD flies mediated by *R4-Gal4*. (**M**–**O**) Systemic glucose levels (**M**), circulating glucose levels (**N**), or circulating trehalose levels (**O**) in control flies or in neuronal-specific *dSLC5A5*-KD flies mediated by *ElaV-Gal4*. In all cases, systemic glucose levels (μg/μL) were normalized to whole-body protein (μg/μL). Results are the mean ± SEM of 30–40 flies analyzed over at least five independent experiments and expressed as the fold change compared with that of the control flies (set at 1.0). Student’s *t*-test was used to derive *p*-values between the transgene control and KD flies. ND, normal diet.

We found a significant reduction in systemic glucose levels in the *MyoIA-Gal4* > *dSLC5A5^RNAi^* or *Cad-Gal4* > *dSLC5A5^RNAi^* flies relative to those in the control flies on an ND (Figure 2A,B). The circulating glucose (Figure 2C) and trehalose (Figure 2D) levels were similarly decreased to a significant extent in the *Cad-Gal4* > *dSLC5A5^RNAi^* flies compared to those in the control flies. In addition, the knockdown of *dSLC5A5* using another independent RNAi line with *MyoIA-Gal4* (*MyoIA-Gal4* > *dSLC5A5^RNAi−1^*) significantly decreased the systemic glucose levels relative to those in the control flies on an ND (Figure S3C). However, the knockdown of *dSLC5A5* in the intestinal stem cells/enteroblasts (using *escargot-Gal4*) or in the enteroendocrine cells (using *Prospero-Gal4*) did not significantly alter systemic glucose levels compared to those in the control flies (Figure 2E,F). Moreover, the knockdown of *dSLC5A5* specifically in the skeletal muscle did not evoke any obvious changes in systemic glucose (Figure 2G), circulating glucose (Figure 2H), or circulating trehalose (Figure 2I) levels relative to those in the control flies, suggesting that the moderate expression of *dSLC5A5* as detected in the skeletal muscles (Figure S1A) does not elicit any obvious effect on normal systemic glucose homeostasis. The targeted knockdown of *dSLC5A5* in the fat body (Figure 2J–L) or neurons (Figure 2M–O) also did not significantly alter systemic glucose or circulating glucose and trehalose levels compared to those in the control flies, which is in agreement with the lack of detectable *dSLC5A5* expression in those tissues (Figure S1A).

### 2.4. Overexpression of dSLC5A5 Alters Systemic Glucose Homeostasis

**Figure 3:**
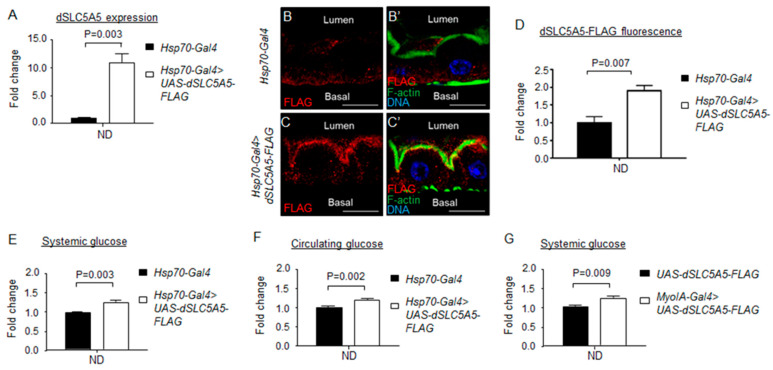
Overexpression of *dSLC5A5* alters systemic glucose metabolism. (**A**) Quantitative real-time PCR analysis of the whole-fly levels of *dSLC5A5* in *Gal4* control flies (*Hsp70-Gal4*) or in whole-body *dSLC5A5*-overexpressing flies mediated by *Hsp70-Gal4* (*Hsp70-Gal4 > dSLC5A5-FLAG*). Flies were subjected to a 40 min heat shock for the induction of dSLC5A5 overexpression. *RPL14* served as an internal control. Results are the mean ± SEM of 15 flies analyzed over three independent experiments per genotype and expressed as the fold change compared with that of the control flies (set at 1.0). Student’s *t*-test was used to derive *p*-values between the control and KD flies. (**B**–**C**′) Representative confocal images of the sagittal sections of midguts (R4c-R5 regions) in (**B**,**B**′) or in whole-body *dSLC5A5*-overexpressing flies mediated by *Hsp70-Gal4* (**C**,**C**′). Flies were subjected to a 40 min heat shock and a 1 h post-recovery (25 °C) regimen for the induction of dSLC5A5 overexpression followed by midgut isolation and immunostaining for FLAG (red), F-actin (green), and DNA (blue). Scale bars represent 10 μm. (**D**) Quantification of FLAG immunofluorescence with the mean intensity for the *Hsp70-Gal4 > dSLC5A5-FLAG* flies normalized to that of control flies (*Hsp70-Gal4*; set at 1.0). For each genotype, three to five intestines were analyzed for FLAG immunofluorescence and the results are represented as the mean fold change ± SEM. (E-G) Systemic glucose levels (E,G) or circulating glucose levels (F) in control flies or in whole-body (**E**,**F**) or midgut-specific (**G**) *dSLC5A5*-overexpressing flies. Systemic glucose levels (μg/μL) were normalized to whole-body protein (μg/μL). Results are the mean ± SEM of 30–40 flies analyzed over at least five independent experiments and expressed as the fold change compared with that of the control flies (set at 1.0). Student’s *t*-test was used to derive *p*-values between the transgene control and *Gal4*-mediated RNAi lines. ND, normal diet.

In reciprocal studies, we assessed the gain-of-function of dSLC5A5 on systemic glucose homeostasis. Previous studies have reported fusing various tags to human SGLT1 for the evaluation of cellular SGLT1 abundance [8]. In the same direction, we fused a FLAG tag to the C-terminus of the full-length dSLC5A5 protein and overexpressed the fusion protein using the *Hsp70-Gal4* driver (*Hsp70-Gal4* > *dSLC5A5-FLAG*) under transient heat shock of 40 min followed by a post-heat shock recovery of 1 h (at 25 °C). This regimen proved effective in inducing transgene expression as we detected an ~10-fold increase in the whole body *dSLC5A5* mRNA level in the *Hsp70-Gal4* > *dSLC5A5-FLAG* flies relative to control flies (Figure 3A). Our results further revealed a stronger FLAG immunosignal in the apical membranes of the enterocytes (marked by the apical membrane marker F-actin) in the *Hsp70-Gal4* > *dSLC5A5-FLAG* flies compared to that in the control flies (Figure 3B,C′). Quantification of the FLAG immunofluorescence showed a near 2-fold increase in the dSLC5A5 content in the enterocyte apical membrane in the *Hsp70-Gal4* > *dSLC5A5-FLAG* flies relative to that in the control flies (Figure 3D). Concomitantly, there was a significant elevation in systemic and circulating glucose levels in the *Hsp70-Gal4* > *dSLC5A5-FLAG* flies relative to those in the control flies (Figure 3E,F).

We further targeted the overexpression of dSLC5A5-FLAG in the midgut using *MyoIA-Gal4* (*MyoIA-Gal4* > *dSLC5A5-FLAG*), which similarly led to an increased accumulation of dSLC5A5-FLAG in the enterocyte apical membranes (marked by the apical membrane marker F-actin) compared to that in the control enterocytes (Figure S4A–D′). The *MyoIA-Gal4* > *dSLC5A5-FLAG* flies also exhibited increased systemic glucose levels compared to those in the control flies (Figure 3G). Overall, our results support a role of dSLC5A5 as a major regulator of systemic glucose homeostasis.

### 2.5. dSLC5A5 Mediates Glucose Uptake in the Midgut

We further posit that dSLC5A5 regulates glucose homeostasis by mediating glucose uptake in the enterocytes. If that is the case, we reason that the loss of dSLC5A5 will abrogate glucose uptake in the midgut and consequently decrease the levels of glucose in the midgut. Indeed, we observed significantly lowered glucose levels in the *Cad-Gal4* > *dSLC5A5^RNAi^* or *MyoIA*-*Gal4* > *dSLC5A5^RNAi^* flies compared to those in the control midguts (Figure S5A,B, leftmost two bars). In another experiment, we found that the *Hsp70-Gal4* > *dSLC5A5-FLAG* flies, upon being subjected to heat shock and a post-recovery regimen, displayed significantly heightened glucose content in their midguts compared to that in the midguts of the heat-shocked control flies (Figure S5C, leftmost two bars). Together, these results provide evidence supporting a role of dSLC5A5 in regulating glucose absorption in the midgut.

To directly assess the glucose uptake function of dSLC5A5, we used a non-metabolized fluorescent glucose analog 2-NBDG as an indicator of glucose uptake in the midgut. We first determined whether 2-NBDG uptake by the midgut cells could be inhibited in the presence of glucose. Indeed, in an ex vivo glucose competition assay, we observed a dose-dependent diminution of 2-NBDG fluorescence in the wild-type (*w^1118^*) enterocytes upon incubation with increasing amounts of glucose (Figure S6A–C). These observations suggest that 2-NBDG and glucose compete for the same glucose transporter to enter the cells, further indicating that 2-NBDG uptake can accurately reflect glucose uptake in the midgut.

**Figure 4:**
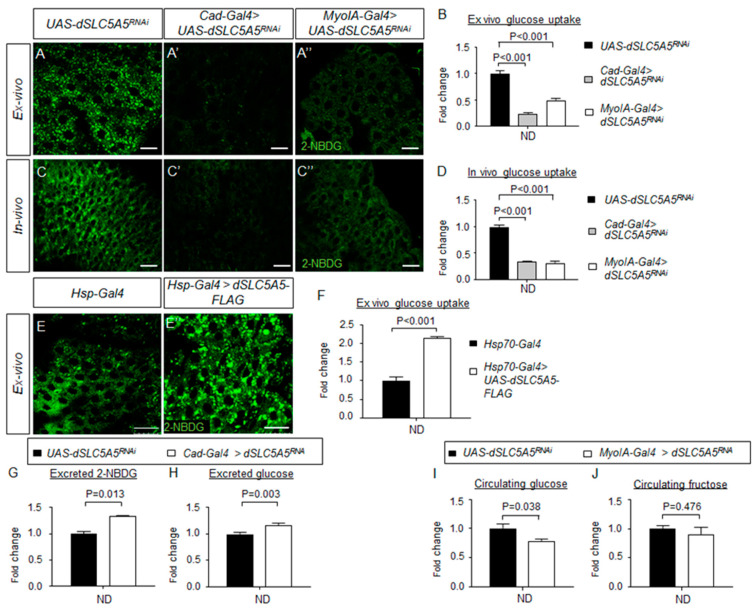
Intestinal-specific inhibition or overexpression of dSLC5A5 disrupts glucose uptake in the enterocytes. (**A**–**C**″) Representative confocal images of the intracellular accumulation of 2-NBDG in the ex vivo (**A**–**A**″) or in vivo (**C**–**C**″) enterocytes in the R4c-R5 midgut region of control flies (*UAS-dSLC5A5^RNAi^*) (**A**,**C**) or in midgut-specific *dSLC5A5*-KD flies mediated by *Cad-Gal4* (**A**′,**C**′) or *MyoIA-Gal4* (**A**″,**C**″). Scale bars represent 20 μm. (**B**,**D**) Quantification of 2-NBDG fluorescence with the mean intensity for the different genotypes normalized to that of control flies (set at 1.0) in ex vivo glucose uptake assay (**B**) or in vivo uptake assay (**D**). For each genotype, three to five intestines were analyzed for 2-NBDG fluorescence and results are represented as the mean fold change ± SEM. (**E**,**E**′) Representative confocal images of the intracellular accumulation of 2-NBDG in the enterocytes of the R4c-R5 midgut region of control flies (*Hsp70-Gal4*) (**E**) or in whole-body *dSLC5A5*-overexpressing flies mediated by *Hsp70-Gal4* (**E**′). Flies were subjected to a 40 min heat shock followed by a 1 h post-recovery regimen (25 °C) for the induction of dSLC5A5 overexpression followed by midgut isolation and 2-NBDG incubation ex vivo. Scale bars represent 20 μm. (**F**) Quantification of 2-NBDG fluorescence with the mean intensity for the *Hsp70-Gal4 > dSLC5A5-FLAG* flies normalized to that of control flies (set at 1.0). For each genotype, three to five intestines were analyzed for 2-NBDG fluorescence and results are represented as the mean fold change ± SEM. (**G**,**H**) 2-NBDG levels (**G**) or glucose levels (**H**) in the excrement of control flies or midgut-specific *dSLC5A5*-KD flies mediated by *Caudal-Gal4* after 2-NBDG or glucose feeding for an hour following overnight starvation. Five 1-week-old female flies per genotype were used for 6 h excrement collection. (**I**,**J**) Circulating glucose levels (**I**) or circulating fructose levels (**J**) in control flies or midgut-specific *dSLC5A5*-KD flies mediated by *MyoIA-Gal4* after glucose or fructose feeding for an hour following overnight starvation. Results are the mean ± SEM of 30–40 flies analyzed over at least five independent experiments and expressed as the fold change compared with that of the control flies (set at 1.0). Student’s *t*-test was used for statistical analysis between the transgene control and *KD* flies. ND, normal diet.

We then used 2-NBDG to evaluate glucose absorption in midguts with genetically altered levels of dSLC5A5. In an ex vivo glucose uptake assay, our results revealed dramatically decreased 2-NBDG fluorescence in the *dSLC5A5*-silenced midguts compared to that in the control midguts (Figure 4A,A″), with 2-NBDG fluorescence reaching only ~25% (for the *Cad-Gal4 > dSLC5A5^RNAi^* midguts) and ~50% (for the *MyoIA-Gal4 > dSLC5A5^RNAi^* midguts) of that of the control midguts (Figure 4B). Similarly, in an in vivo glucose uptake assay, we observed that 2-NBDG fluorescence was drastically decreased in the *dSLC5A5*-silenced midguts compared to that in the control midguts (Figure 4C–C″), with 2-NBDG fluorescence reaching only 30%–35% (for both *Cad-Gal4 > dSLC5A5^RNAi^* and *MyoIA-Gal4 > dSLC5A5^RNAi^* midguts) of that in the control midgut (Figure 4D). However, in the midguts of the *Hsp70-Gal4 > dSLC5A5-FLAG* flies subjected to heat shock and a post-recovery regimen, the 2-NBDG fluorescence intensity was increased (up to 2-fold) compared to that in the control flies (Figure 4E,E′,F). These results indicate that dSLC5A5 positively regulates glucose uptake in the midgut on an ND.

Dietary nutrients not absorbed by the enterocytes invariably get expelled from the body through the excreta. We therefore assessed 2-NBDG fluorescence levels in the excreta of starved *dSLC5A5*-silenced flies and control flies that were fed transiently with 2-NBDG. A significant increase in 2-NBDG fluorescence levels in the excreta of the *Cad-Gal4 > dSLC5A5^RNAi^* flies was detected compared to those in control flies (Figure 4G). We then directly quantified the amount of glucose in the excreta and similarly found a significantly higher glucose levels in that of the *Cad-Gal4 > dSLC5A5^RNAi^* flies relative to those of the control flies (Figure 4H). In all, these observations are consistent with the role of dSLC5A5 acting as a glucose transporter in the midgut.

Next, we probed whether dSLC5A5 might also be involved in fructose transport in the midgut, by feeding flies with exclusively either glucose or fructose followed by analysis of their circulating glucose or fructose levels, respectively. Our results showed that whereas the circulating glucose levels were decreased significantly in the *MyoIA-Gal4 > dSLC5A5^RNAi^* flies relative to those in the control flies fed with glucose (Figure 4I), the circulating levels of fructose remained comparable between both groups of flies fed with fructose (Figure 4J). These results point to a specific role of dSLC5A5 as a glucose transporter in the midgut.

### 2.6. dSLC5A5 Undergoes Dynamin-Dependent Endocytosis in Midgut Enterocytes

**Figure 5:**
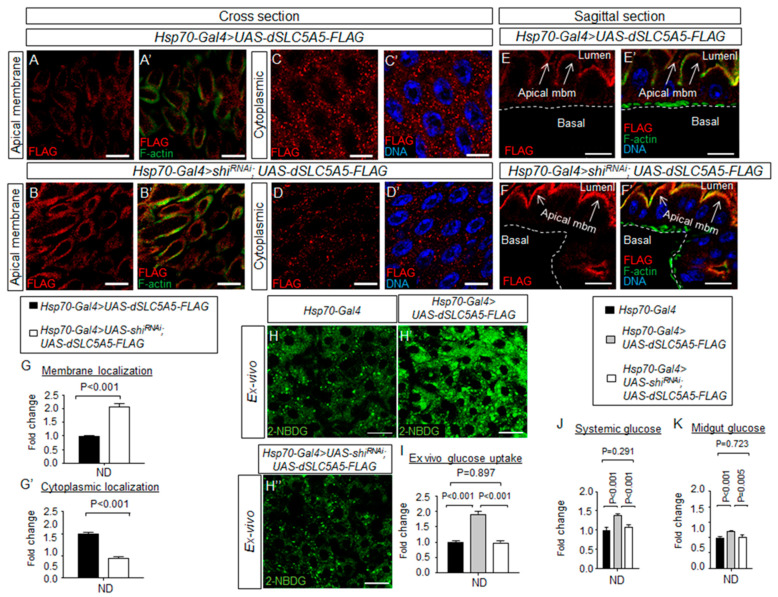
Dynamin-dependent endocytosis regulates dSLC5A5 membrane trafficking and glucose uptake in the enterocytes. (**A**–**F**′) Representative confocal images of dSLC5A5-FLAG-overexpressing enterocytes in the R4c-R5 midgut region that are immunostained for FLAG (red), F-actin (green), and DNA (blue) in the absence (**A**,**A**′,**C**,**C**′,**E**,**E**′) or the presence (**B**,**B**’,**D**,**D**′,**F**,**F**′) of *shibire*-KD mediated by *Hsp70-Gal4*. Scale bars represent 20 μm. (**G**,**G**′) Quantification of the FLAG immunofluorescence in the apical membrane (**G**) or cytoplasm (**G**′) of enterocytes with the mean intensity for the *Hsp70-Gal4 > shibire^RNAi^*; *dSLC5A5-FLAG* flies normalized to that of control flies (*Hsp70-Gal4*; set at 1.0). Data represent the mean fold change ± SEM from three independent images. (**H**–**H**″) Representative confocal images of the intracellular accumulation of 2-NBDG in the enterocytes of the R4c-R5 midgut region of control flies (*Hsp70-Gal4*), dSLC5A5-FLAG-overexpressing flies (*Hsp70-Gal4 > dSLC5A5-FLAG*) or dSLC5A5-overexpressing and *shibire*-KD flies (*Hsp70-Gal4 > shibire^RNAi^*; *dSLC5A5-FLAG*). The flies were subjected to a 40 min heat shock followed by a 1 h post-recovery regimen (25 °C) for the induction of dSLC5A5 overexpression followed by midgut isolation and 2-NBDG incubation ex vivo. Scale bars represent 20 μm. (**I**) Quantification of 2-NBDG fluorescence with the mean intensity for the different genotypes normalized to that of driver control flies (*Hsp70-Gal4*; set at 1.0). For each genotype, three to five intestines were analyzed for 2-NBDG fluorescence and results are represented as mean fold change ± SEM. (**J**,**K**) Systemic glucose levels (**J**) or midgut glucose levels (**K**) in control flies (*Hsp70-Gal4*), dSLC5A5-FLAG-overexpressing flies (*Hsp70-Gal4 > dSLC5A5-FLAG*), or dSLC5A5-overexpressing and *shibire*-KD flies (*Hsp70-Gal4 > shi^RNAi^*; *dSLC5A5-FLAG*). Systemic glucose levels (μg/μL) were normalized to whole-body protein (μg/μL). Results are the mean ± SEM of 30–40 flies analyzed over at least five independent experiments and expressed as the fold change compared with that of the control flies (set at 1.0). Student’s *t*-test was used for statistical analysis. ND, normal diet.

Given their important roles in governing nutrient uptake or secretion, nutrient transporters, including glucose transporters, are highly regulated in cells by processes such as endocytosis [20]. We therefore asked whether dSLC5A5 might be subjected to endocytic regulation in the enterocyte surface membranes. One essential protein for endocytosis is the GTPase dynamin, which was first cloned from the *shibire* temperature-sensitive paralytic *Drosophila* mutant [21]. We abrogated the dynamin function by using the *Hsp70-Gal4* to globally knock down *shibire* and simultaneously overexpress *dSLC5A5-FLAG* in flies (*Hsp70-Gal4 > shi^RNAi^;UAS-dSLC5A5-FLAG*). Following heat shock and a post-recovery regimen, midguts were isolated and immunostained for FLAG (red, dSLC5A5 amounts) and F-actin (green, enterocyte surface membrane). Interestingly, we observed an increased abundance of dSLC5A5 in the surface membranes of the enterocytes in the *Hsp70-Gal4 > shi^RNAi^;UAS-dSLC5A5-FLAG* flies compared to that in the control *Hsp70-Gal4 > UAS-dSLC5A5-FLAG* flies in both cross-sectional (Figure 5A,B′) and longitudinal-section (Figure 5E,F′) views of the midgut. In parallel, the cytoplasmic level of dSLC5A5 in the enterocytes of the *Hsp70-Gal4 > shi^RNAi^;UAS-dSLC5A5-FLAG* flies was decreased compared to that in the control flies (Figure 5C–D′). Quantification of the FLAG immunosignal further indicates a significant 2-fold increase in the dSLC5A5 surface membrane concentration and a significant 0.5-fold reduction in the dSLC5A5 cytoplasmic concentration in the *Hsp70-Gal4 > shi^RNAi^;UAS-dSLC5A5-FLAG* enterocytes compared to that in the control enterocytes (Figure 5G,G′). These results indicate that dynamin-dependent endocytosis is an important mechanism in regulating the plasma membrane abundance of dSLC5A5 in midguts.

To further probe whether dynamin-dependent endocytosis of dSLC5A5 affects dSLC5A5-mediated glucose absorption, we examined the effect of knocking down *shibire* on 2-NBDG uptake in the enterocytes. As expected, there was an increase in the 2-NBDG fluorescence intensity (~1.8-fold) in the *Hsp70-Gal4 > UAS-dSLC5A5-FLAG* midguts relative to that in the control *Hsp70-Gal4* midguts following heat shock and a post-recovery regimen (Figure 5H,H′,I; see also Figure 4E,E′,F). Such an increase, however, was suppressed in the *Hsp70-Gal4 > shi^RNAi^; UAS-dSLC5A5-FLAG* midguts (Figure 5H′,H″,I). Concomitantly, the heightened systemic (Figure 5J) and intestinal (Figure 5K) glucose levels in the *Hsp70-Gal4 > UAS-dSLC5A5-FLAG* flies were abolished in the *Hsp70-Gal4 > shi^RNAi^;UAS-dSLC5A5-FLAG* flies on an ND. These results indicate that impairment of dynamin-mediated endocytosis suppresses glucose absorption in the enterocytes, despite the entrapping of dSLC5A5 in the enterocyte apical membrane (Figure 5A–B′,E–F′).

### 2.7. dSLC5A5 Intracellular Trafficking Is Involved in the Short-Term Regulation of dSLC5A5 by Glucose

**Figure 6:**
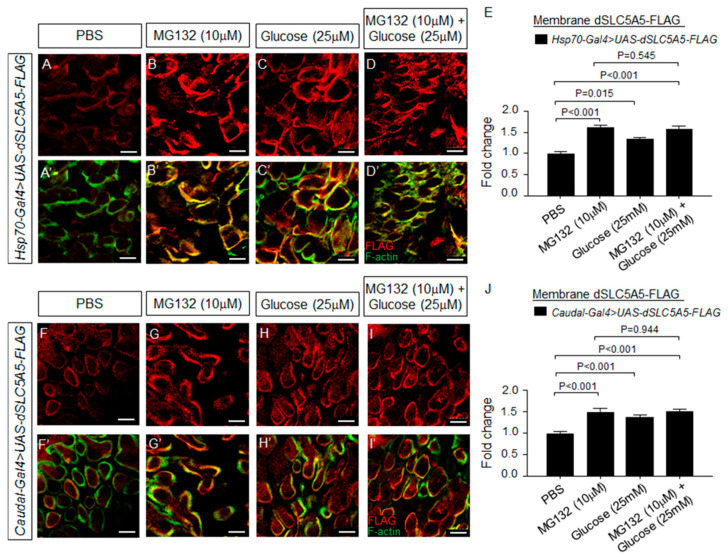
Regulation of dSLC5A5 membrane abundance by proteasome inhibitor, high glucose, or both. (**A**–**D**′) Representative confocal images of the *Hsp70-Gal4*-mediated dSLC5A5-FLAG-overexpressing enterocytes in the R4c-R5 midgut region that are immunostained for FLAG (red), F-actin (green), and DNA (blue). Flies were subjected to a 40 min heat shock followed by a 1 h post-recovery session (25 °C) for the induction of dSLC5A5 overexpression followed by midgut isolation and 30 min of incubation with PBS (**A**,**A**′), proteasome inhibitor MG132 (10 µM) (**B**,**B**′), high extracellular glucose (25 mM) (**C**,**C**′), or a combination of these (**D**,**D′**). Scale bars represent 10 μm. (**E**) Quantification of the apical membrane FLAG immunofluorescence with the mean intensity for the different genotypes normalized to that of PBS treatment (set at 1.0). For each genotype, three to five intestines were analyzed for FLAG immunofluorescence and results are represented as the mean fold change ± SEM. (**F**–**I**′). Representative confocal images of *Cad-Gal4*-mediated dSLC5A5-FLAG-overexpressing enterocytes in the R4c-R5 midgut region that are immunostained for FLAG (red), F-actin (green), and DNA (blue). Flies were subjected to a 40 min heat shock followed by a 1 h post-recovery session (25 °C) for the induction of dSLC5A5 overexpression followed by midgut isolation and 30 min of incubation with PBS (**F**,**F**′), proteasome inhibitor MG132 (10 µM) (**G**,**G**′), high extracellular glucose (25 mM) (**H**,**H**′), or a combination of these (**I**,**I**′). Scale bars represent 10 μm. (**J**) Quantification of the apical membrane FLAG immunofluorescence with the mean intensity for the different genotypes normalized to that of PBS treatment (set at 1.0). For each genotype, three to five intestines were analyzed for FLAG immunofluorescence and results are represented as the mean fold change ± SEM.

We further probed the downstream fates of dSLC5A5 subsequent to its endocytosis from the enterocyte cell surface. It is possible that dSLC5A5 could be targeted to the lysosomes for enzymatic proteolysis or recycled back to the plasma membrane for reuse, as seen for SGLT1 in cultured cells [8]. These two targeting mechanisms are intimately linked, in that blockade of the lysosomal degradation pathway by proteasome inhibitors [22] could promote recycling back to the membrane [8]. Indeed, when we inhibited lysosomal degradation in the dSLC5A5-FLAG-overexpressing midguts with the proteasome inhibitor MG132, we observed an enhancement of dSLC5A5-FLAG levels in the enterocyte apical membrane (marked by F-actin) relative to the PBS-treated midgut enterocytes for both *Hsp70-Gal4 > dSLC5A5-FLAG* (Figure 6A,B′) and *Cad-Gal4 > dSLC5A5-FLAG* (Figure 6F,G′) flies. Quantification of the apical membrane FLAG immunofluorescence level revealed an ~1.5-fold increase in the dSLC5A5-FLAG levels in the MG132-treated enterocytes compared to those in PBS-treated enterocytes (Figure 6E,J). These results support the notion that intracellular lysosomal proteolysis and plasma membrane recycling are involved in maintaining dSLC5A5 homeostasis in the enterocytes.

The abundance of nutrient transporters in the plasma membrane is tightly regulated by substrate availability. We therefore interrogated the regulation of dSLC5A5 by glucose in the midgut. When transiently exposed to high glucose, dSLC5A5-FLAG abundance in the apical membrane (marked by F-actin) was increased relative to the PBS-treated enterocytes for both *Hsp70-Gal4 > dSLC5A5-FLAG* (Figure 6A,A′,C,C′) and *Cad-Gal4 > dSLC5A5-FLAG* (Figure 6F,F′,H,H′) flies. Quantification of the apical membrane FLAG immunofluorescence level revealed a significant, close to 1.5-fold, increase in the dSLC5A5-FLAG level in the glucose-treated enterocytes compared to their PBS-treated counterparts (Figure 6E,J). Therefore, like intestinal SGLT1 [23,24,25,26], glucose induces short-term upregulation of dSLC5A5 in the midgut. Interestingly, co-incubation of the dSLC5A5-FLAG-overexpressing midguts with MG132 and high glucose does not further increase the dSLC5A5-FLAG density in the enterocyte surface membranes compared to treatment with either alone, for both *Hsp70-Gal4 > dSLC5A5-FLAG* midguts (Figure 6D,D′,E) and the *Cad-Gal4 > dSLC5A5-FLAG* midguts (Figure 6I,I′,J). These observations suggest that dSLC5A5 intracellular trafficking is involved in the short-term regulation of dSLC5A5 by glucose.

### 2.8. dSLC5A5 Is a Critical Determinant of Systemic Glucose Homeostasis under a High-Sugar-Diet Condition

Our results have demonstrated an important role of dSLC5A5 in maintaining normal glucose homeostasis (Figure 1, Figure 2 and Figure 3). We therefore further asked whether dSLC5A5 is also involved in the regulation of glucose homeostasis under a high-sugar-diet (HSD) condition. For that, we developed an HSD feeding regimen (Figure S7A) that led to an increase in the systemic glucose levels and circulating glucose and trehalose levels in wild-type *w^1118^* flies (Figure S7B–D). The HSD-fed flies also developed insulin resistance, as shown by the reduced phosphorylation of Akt in response to insulin (Figure S7E), as well as obesity, as indicated by increased lipid storage in the fat body adipocytes (Figure S7F,F′). Together, these observations indicate that the HSD condition leads to dysregulation of glucose metabolism in flies. Furthermore, there was a robust increase in the transcript level of *dSLC5A5* in the midgut (by ~3-fold, Figure S8A) on an HSD that was not detected in other tissues, including the skeletal muscle (Figure S8B) and the fat body (Figure S8C), suggesting that dSLC5A5 is primarily induced in the midgut by an HSD.

**Figure 7:**
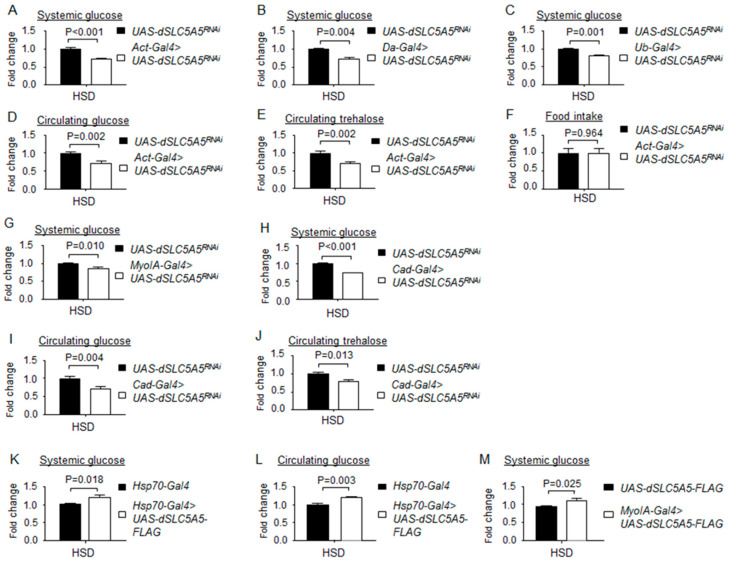
Loss- or gain-of-function of dSLC5A5 alters systemic glucose metabolism on a high-sugar diet (HSD). (**A**–**C**) Systemic glucose levels in control flies (*UAS-dSLC5A5^RNAi^*) or in flies bearing the whole-body KD of *dSLC5A5* using different ubiquitous drivers, *Act-Gal4* (**A**), *Da-Gal4* (**B**), or *Ub-Gal4* (**C**). (**D**,**E**) Circulating levels of glucose (**D**) and trehalose (**E**) in control flies or in flies with the whole-body KD of *dSLC5A5* mediated by *Act-Gal4* (*Act-Gal4 > dSLC5A5^RNAi^*). (**F**) Food consumption in control flies or in flies with the whole-body KD of *dSLC5A5* mediated by *Act-Gal4* (*Act-Gal4 > dSLC5A5^RNAi^*). (**G**,**H**) Systemic glucose levels in control flies or in midgut-specific *dSLC5A5*-KD flies mediated by *MyoIA-Gal4* (**G**) or *Cad-Gal4* (**H**) on an HSD. (**I**,**J**) Circulating glucose levels (**I**) or circulating trehalose levels (**J**) in control flies or in midgut-specific *dSLC5A5*-KD flies mediated by *Cad-Gal4*. (**K**–**M**) Systemic glucose levels (**K**), circulating glucose levels (**L**), or systemic glucose levels (**M**) in control flies (*Hsp70-Gal4*) or dSLC5A5-FLAG-overexpressing flies (*Hsp70-Gal4 > dSLC5A5-FLAG*) flies. In all cases, systemic glucose levels (μg/μL) were normalized to whole-body protein (μg/μL). Results are the mean ± SEM of 30–40 flies analyzed over at least five independent experiments and are expressed as the fold change compared with that of the control flies (set at 1.0). Student’s *t*-test was used to derive *p*-values between the driver control and KD flies. HSD, high-sugar diet.

Next, to determine the functional implications of the HSD-mediated induction of dSLC5A5, we knocked down *dSCL5A5* under HSD condition with the ubiquitous *Act-*, *Da-*, or *Ub-Gal4* drivers and observed a significant decrease in systemic glucose levels in the knockdown flies compared to those in the control flies (Figure 7A–C). The circulating glucose and circulating trehalose levels were also significantly decreased in the *Act-Gal4 >* *dSLC5A5^RNAi^* flies compared to those in the control flies on an HSD (Figure 7D,E). Food intake remained comparable between the *Act-Gal4 >* *dSLC5A5^RNAi^* flies and the control flies on an HSD (Figure 7F). We further targeted the knockdown of *dSLC5A5* in the midgut with *MyoIA-Gal4* or *Cad-Gal4* and observed a significant reduction in systemic glucose levels (Figure 7G,H), circulating glucose and circulating trehalose levels (Figure 7I,J), and midgut glucose levels (Figure S5A,B, rightmost two bars) relative to those in the control flies on an HSD. Conversely, overexpression of dSLC5A5 in whole flies (using *Hsp70-Gal4* followed by heat shock and a post-recovery regimen) or specifically in the midgut (using *MyoIA-Gal4*) significantly increased systemic and circulating glucose levels (Figure 7K,M) and midgut glucose levels (Figure S5C, rightmost two bars) relative to those in the control flies on an HSD. In contrast, flies with neuronal-targeted (using *Elav-Gal4*), fat-body-targeted (using *R4-Gal4*), or muscle-targeted (using *Mhc-Gal4*) knockdown of *dSLC5A5* displayed systemic and circulating glucose and circulating trehalose concentrations comparable with those of the control flies on an HSD (Figure S9A,I). Taken together, these results indicate that dSLC5A5 plays a predominant role in the midgut in determining HSD-mediated glucose metabolic responses.

## 3. Discussion

In this study, we report the first identification of a dietary glucose transporter in the *Drosophila* midgut. Our results indicate that dSLC5A5 is expressed in the enterocyte apical membrane and acts in the enterocytes to direct glucose uptake, which modulates systemic glucose homeostasis. Given that the *MyoIA-Gal4* is an enhancer trap in brush border *Myosin IA* that drives expression specifically in the midgut enterocytes [27,28] whereas *Cad-Gal4* drives expression in all epithelial cells, including the enterocytes, within the R4c-R5 region of the midgut [29,30], our results suggest that dSLC5A5 is expressed in and acts in the midgut enterocytes to maintain normal systemic glucose homeostasis. Moreover, although dSLC5A5 has been previously reported to be expressed in the intestinal stem cells [31], our results indicate that dSLC5A5 does not act in these cells to regulate systemic glucose homeostasis. We further show that dSLC5A5 undergoes dynamin-dependent endocytosis in the enterocyte apical membrane, which is contrary to a previous report that SGLT1 undergoes apical endocytosis in cultured non-enterocyte cell lines in a manner independently of dynamin [8]. We also demonstrate for the first time the functional consequence of apical endocytosis for the glucose uptake ability of glucose transporter in the enterocyte. Finally, we reveal that while glucose upregulates the dSLC5A5 level in the fly midgut that is similar to that observed for intestinal SGLT1, the upregulation of dSLC5A5 by glucose involves the alteration of dSLC5A5 intracellular trafficking subsequent to its endocytosis, which is a previously unknown aspect of glucose transporter regulation by glucose. In all, our work uncovers new mechanisms that govern the regulation of glucose transporter function and activity in the enterocyte apical membranes.

Using the in vivo *Drosophila* midgut system, we demonstrated that the genetic inhibition of shibire, the *Drosophila* homolog of dynamin, entraps dSLC5A5 in the apical membranes of the enterocytes. However, a previous study reported that the overexpression of a dominant-negative form of dynamin in cultured cells appears not to alter the plasma membrane levels of SGLT1 [8], suggesting that dynamin does not play a significant role in the apical endocytosis of SGLT1. As this finding was obtained from experiments that were performed in a cell line (human embryonic kidney cells) that is not a physiologically relevant model for human enterocytes, it might not accurately reflect the endogenous role of dynamin in glucose transporter endocytosis in the intestinal enterocytes. While the differing data on the role of dynamin between SGLT1 and dSLC5A5 endocytosis could lie in the different cellular systems used and the different approaches used to lower dynamin levels, our findings provide the first evidence supporting an essential role of dynamin in the endocytosis of glucose transporter in a physiological context, which serves as the basis for future studies aimed at elucidating the endogenous function of dynamin in the apical endocytosis of SGLT1 in the small intestine.

What is the functional consequence of endocytosis for glucose transporter activity in enterocytes? Our results show that the inhibition of dSLC5A5 endocytosis is associated with a decrease in glucose uptake in the enterocytes, even though dSLC5A5 abundance in the apical membrane is increased. This interesting finding indicates that the endocytosis of dSLC5A5 is required for the ability of dSLC5A5 to transport glucose. It is therefore possible that dSLC5A5 in the apical membrane needs to be refreshed via endocytosis to maintain its glucose uptake ability. The retrieval of the transport-inactive dSLC5A5 from the apical membrane via endocytosis would then allow for newly synthesized dSLC5A5 to be incorporated into the apical membrane. Alternatively, the endocytosed dSLC5A5 could undergo re-activation in the endosomes before being recycled back to the membrane, in a mechanism that is similar to those involved in the regulation of cell surface receptors, such as G protein-coupled receptors [32] and cytokine receptors [33]. In either case, when endocytosis is abolished, as in our case with the *shibire*-silenced enterocytes, the ability of dSLC5A5 to be replenished is correspondingly abrogated, culminating in an accumulation of transport-inactive dSLC5A5 in the apical membrane that is paralleled by a decrease in the uptake of glucose in the enterocytes.

Glucose is an important regulator of intestinal glucose transporter expression, as documented by an upregulation in SGLT1 transcription in the jejunum brush border membrane of rats fed on a high-carbohydrate diet for several days [34], an increase in SGLT1 protein content in the small intestine of mice after glucose gavage [23], and an enhancement in glucose uptake in the brush border membrane after rapid exposure of the rat jejunal mucosa to glucose [24,25,26]. Our work here similarly found that dSLC5A5 transcription in the *Drosophila* midgut was induced in response to high dietary sugar intake and that the dSLC5A5 protein level in the apical membranes was increased by acute high glucose exposure. The regulation of glucose transporter by glucose appears to critically involve the intracellular trafficking mechanisms of the glucose transporter, including the promotion of the SGLT1 exocytosis [35]. In our study, we observed that in the presence of high glucose, the concomitant inhibition of lysosomal degradation by a proteasome inhibitor such as MG132 does not further increase the dSLC5A5 level in the enterocyte apical membrane, suggesting that the abrogation of this pathway in the presence of glucose only minimally impacts the level of dSLC5A5 in the enterocyte apical membrane. Potentially, glucose could entrap dSLC5A5 in the apical membrane by blunting its endocytosis or by promoting the recycling of dSLC5A5 from the endosomes to the apical membrane. Either circumstance is likely to lead to decreased amounts of dSLC5A5 being targeted to the lysosomal degradation pathway, which lowers the contribution of this intracellular trafficking pathway to dSLC5A5 homeostasis in the apical membrane. Further elucidation of such new mechanisms of intestinal glucose transporter regulation by glucose would expand our understanding of SGLT1 regulation in the small intestine.

**Figure 8:**
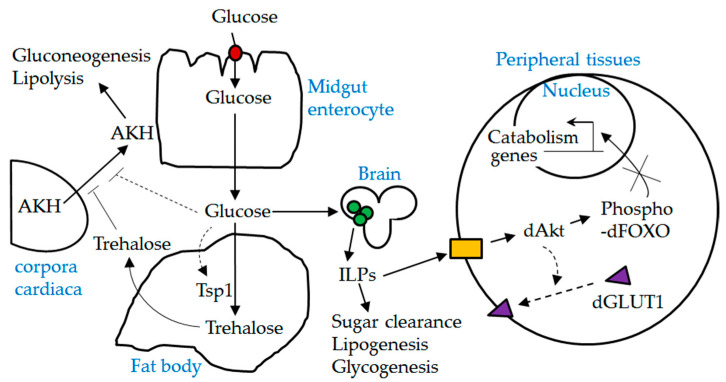
A schematic diagram depicting the interplay between dSLC5A5 and other players involved in glucose homeostasis. Red solid circle, dSLC5A5; green solid circles, insulin producing cells (IPCs); ILPs, insulin-like peptides; yellow solid box, *Drosophila* insulin-like receptor (dInR); AKH, adipokinetic hormone. Dotted lines indicate possible mechanisms. dSLC5A5-mediated uptake of glucose in the midgut into the circulation is expected to stimulate the release of ILPs from the IPCs while suppressing the secretion of AKH from the corpora cardiaca. Upon their release, ILPs act through the dInR to elicit the phosphorylation of dFOXO, which leads to the cytoplasmic retention and inactivation of dFOXO. It is also possible that activation of dInR signaling promotes the plasma membrane localization of dGLUT1.

In summary, we present in vivo evidence supporting the role of dSLC5A5 in midgut glucose uptake. It will be interesting to examine the interplay between dSLC5A5 and other players of glucose homeostatic mechanisms in future studies (see also Figure 8)

## 4. Materials and Methods

### 4.1. Fly Stocks and Transgenic Flies

All fly stocks were maintained at 25 °C on standard medium unless otherwise stated. The following stains were used in this study: *w^1118^* (BDSC BL6326), *Act-Gal4* (BDSC BL3954), *Da-Gal4* (BDSC BL55851), *Ub-Gal4* (BDSC BL32551), *Caudal* (*Cad*)*-Gal4* (BDSC BL3042), *R4-Gal4* (BDSC BL33832), *ElaV-Gal4* (BDSC BL458), *Mhc-Gal4* (BDSC BL38464), *Hsp70-Gal4* (BDSC BL2077), *Escargot-Gal4* (Kyoto DGRC 114-042), *Prospero-Gal4* (BDSC 80572), *UAS-dSLC5A5^RNAi^* (BDSC BL63568), *UAS-dSLC5A5^RNAi^* (VDRC 40650), and *UAS-shibire^RNAi^* (BDSC BL28513). The *MyoIA-Gal4* fly line is a gift from Dr. Bruce Edgar (University of Utah, Salt Lake City, UT, USA). The *dSLC5A5-FLAG* clone (UFO11244) was obtained from the *Drosophila* Genomics Resource Center (DGRC) and contains full-length *dSLC5A5* cDNA with a C-terminus FLAG HA tag in a pUAST vector. *UAS-dSLC5A5-FLAG* transgenic flies were generated by BestGene Inc. (Chino Hills, CA, USA). For each cross, the *Gal4* or *UAS-RNAi* progenies derived from the cross are used as controls.

### 4.2. Glucose Assay

One-week-old flies were analyzed. Whole flies (4 males and 4 females per genotype) or intestines dissected from 12 female flies of each genotype were homogenized in 100 μL of PBST (0.05% Triton X-100) with the protease inhibitor Cocktail (Sigma, St. Louis, MO, USA, P2714). After a 10 min centrifugation (13,000 rpm, 4 °C), 2 μL of the supernatant was used to detect the protein concentration (Bradford assay) and the remaining supernatant was heated for 10 min at 70 °C, followed by 3 min of centrifugation (13,000 rpm 4 °C). From the heated supernatant, 20 μL was diluted in 80 μL distilled water (DW) and incubated with 900 μL of Glucose Reagent (Thermo Scientific, #TR15421, Waltham, MA, USA) for 3 min at 37 °C. The absorbance of 340 nm was then measured, and the glucose content was calculated with a glucose standard curve. The final glucose concentration (μg/μL) was obtained by normalizing the content to the protein concentration.

### 4.3. Hemolymph Trehalose Assay

One-week-old flies were analyzed, and 30–40 female flies were used for hemolymph collection. Flies punched in the thorax by needles (27G ½″) were transferred to a 0.5 mL microfuge tube that has a small hole at the bottom and is filled with a layer of glass wool (Sigma; 20411, St. Louis, MO, USA). The 0.5 mL tube was then placed into a 1.5 mL microfuge tube to be centrifuged at 9000× *g* for 5 min at 4 °C. Then, 1 μL of the hemolymph was carefully removed from the collection tube and diluted in 99 μL of trehalase buffer (TB) (5 mM Tris pH 6.6137 mM NaCl, 2.7 mM KCl), to be immediately heated at 70 °C for 10 min to inactivate the endogenous trehalase. Out of each heat-treated sample, 10 μL was mixed with 90 μL of TB while another 10 μL was added into 90 μL of TB with trehalase (TS) (10 μL of trehalase (Sigma; T8778-1UN, St. Louis, MO, USA) in 1 mL of trehalase buffer) in order to convert trehalose to glucose. After 24 h incubation at 37 °C, glucose contents in TB and TS samples were detected with Glucose Reagent (Thermo Scientific, Waltham, MA, USA, #TR15421). The additional amount of glucose in the TS sample compared to the TB sample was considered to be digested from trehalose.

### 4.4. Nile Red Staining

Nile Red staining was performed as modified from previously described [36]. Briefly, the fat bodies dissected from 1-week-old flies were fixed with 3.7% formaldehyde for 20 min at room temperature and then treated with 5 μg/μL of Nile Red solution for 20 min. After four washes (15 min per wash) with PBST (0.1% Triton X-100), tissues were mounted in Vectashield media (Vector Laboratories, Inc., Burlingame, CA, USA) and imaged at 40X magnification using the Olympus FV1000 confocal microscope.

### 4.5. Immunostaining

The midguts were dissected from 1-week-old flies in PBS and fixed with 10% formaldehyde for 10 min at room temperature. The fixed tissues were then subjected to three rounds of wash (15 min per wash) with 0.1% PBST (0.1% Triton X-100 in PBS) and blocked with 5% donkey serum (Jackson ImmunoResearch Laboratories, West Grove, PA, USA 107-000-121) for 1 h at room temperature followed by incubation with primary antibodies in 5% donkey serum at 4 °C. The tissues were then washed three times, 10 min per wash, in 0.1% PBST, followed by 2 h incubation with Alexa Fluor conjugated secondary antibodies (Jackson Immunoresearch Laboratories, West Grove, PA, USA) in 0.1% PBST at room temperature. After washing as before, the tissues were mounted in Vectashield media (Vector Laboratories, Inc., Burlingame, CA, USA) and viewed under a laser scanning confocal microscope (Olympus FV1000, Tokyo, Japan).

The following reagents were used for immunostaining: mouse anti-FLAG (1:500, Sigma (F1804)), Alexa Fluor 488 phalloidin (10 μM, Invitrogen, Waltham, CA, USA, A12379), donkey anti-mouse Cy3 (1:150, Jackson ImmunoResearch Laboratories), and donkey anti-rat Cy3 (10 μM, Jackson ImmunoResearch Laboratories).

### 4.6. Heat Shock and Post-Recovery Regimen

One-week-old flies were analyzed. Flies of the genotype *Hsp70-Gal4 > dSLC5A5-FLAG* and their respective driver (*Hsp70-Gal4*) and transgene (*dSLC5A5-FLAG*) control flies were incubated at 37 °C for 40 min followed by a 1 h post-recovery period at 25 °C before being subjected to the various assays.

### 4.7. Short-Term Ex Vivo Treatments of Midguts

Abdomens of 1-week-old female flies were cut open and the intestines were dissected in ice-cold PBS. A horizontal incision was made to the posterior midgut with the tip of needles (27G ½″) to expose the enterocytes. Samples were incubated with PBS, PBS with 10 uM MG132 (Sigma; M8699), PBS with 25 mM glucose, or PBS with 10 uM MG132 and 25 mM glucose for 30 min at room temperature. The intestines were then washed briefly three times with PBS and fixed in 10% formaldehyde for 10 min at room temperature before being incubated with anti-FLAG antibody or Alexa Fluor 488 phalloidin, as described above.

### 4.8. Ex Vivo Glucose Uptake Assay

Following 6 h of starvation of 1-week-old female flies with deionized water only, their intestines were dissected in ice-cold PBS. A horizontal incision was made to the posterior midgut with the tip of needles (27G ½″) to expose the enterocytes. Samples were incubated with 300 µM 2-NBDG for 45 min at room temperature. After three brief washes with PBS, tissues were fixed in 3.7% formaldehyde for 15 min, followed by three 5 min washes with PBS. Intestines were mounted in Vectashield media.

### 4.9. In Vivo Glucose Uptake Assay

Following 6 h starvation with deionized water only, 1-week-old female flies were fed with 2-NBDG (750 μM in PBS) for 45 min at room temperature. Their intestines were then dissected in ice-old PBS, fixed in 3.7% formaldehyde for 15 min, washed in PBS for 3 times (5 min per wash), and mounted in Vectashield media.

### 4.10. Ex Vivo Glucose Competition Assay

Following 6 h starvation of 1-week-old female wild-type w^1118^ flies with deionized water only, their midguts were isolated and incubated with 300 μM 2-NBDG and increasing concentrations of glucose followed by the analysis of 2-NBDG fluorescence in the enterocytes.

### 4.11. Excrement Glucose Assay

Following overnight starvation with only deionized water, 1-week-old female flies (*n* = 5 per genotype) were fed with 2-NBDG (250 µM in PBS with blue dye) or ND with 5% blue dye for 1 h at room temperature. The flies were then transferred into a 1.5 mL Eppendorf tube for excrement collection at room temperature for 6 h. After discarding the flies, 100 μL of PBS was added into each tube and the tubes were vortexed to dissolve all the blue-stained excrement deposited in the tube. For 2-NBDG feeding, the absorbance of the blue dye (OD630) and 2-NBDG (488 nm) were measured with the Synergy™ Neo2 Multi-Mode Microplate Reader. For blue ND feeding, 10 µL of the excrement was diluted with PBS (1:20) before blue dye measurement (OD630) and the rest of the excreta (90 μL) was added to 110 µL of Glucose Reagent (Thermo Scientific, Waltham, CA, USA #TR15421) and incubated for 3 min at 37 °C for glucose-level measurement. Final 2-NBDG or glucose content in the excrement was obtained by normalizing the read-out to blue dye amount.

### 4.12. Glucose Feeding Assay

One-week-old female flies (*n* = 30–40 for each genotype) were fed with D-glucose (100 mM in PBS) for 1 h at room temperature following overnight starvation. The hemolymph (I μL) was collected and diluted in 99 μL of PBS and heated at 70 °C for 10 min. After heating, 20 μL of the diluted mixture was incubated with 980 μL of Glucose Reagent (Thermo Scientific, #TR15421, Waltham, USA) for 3 min at 37 °C. Readings at 340 nm absorbance were taken, and the glucose content Cg_0_ (μg/μL) was calculated using a glucose standard curve. The circulating glucose level equals 5 × Cg_0_ (μg/μL).

### 4.13. Fructose Feeding Assay

One-week-old female flies (*n* = 30–40 for each genotype) were fed with fructose (20 mg/mL in PBS) for 1 h at room temperature following overnight starvation. The hemolymph (I μL) was collected and diluted in 99 μL of PBS and heated at 70 °C for 10 min. After heating, 20 μL of the diluted mixture was incubated with 980 μL Glucose Reagent (Thermo Scientific, #TR15421, Waltham, MA, USA) and 0.2 μL of glucose-6-phosphate isomerase (PGI, Roche, Branchburg, NJ, USA, #10127396001), while another 20 μL was incubated with 980 μL Glucose Reagent (Thermo Scientific, #TR15421) only. After 3 min of incubation at 37 °C, the glucose level of the mixture with PGI (Cg) or without PGI (Cg_0_) was calculated based on absorbance of 340 nm using a glucose standard curve. The circulating fructose level (μg/μL) was calculated based on the equation:

∆Cg = Cg − Cg_0_ (1)

### 4.14. Real-Time Quantitative PCR (qPCR)

Total RNA was extracted from tissues in 1-week-old flies (*n* = 15 per genotype) using Trizol Reagent (Ambion, Austin, TX, USA). cDNA using oligo(dT) was synthesized from total RNA (500 ng to 1 μg each) with Superscript III reverse transcriptase (Invitrogen). qPCR amplification reactions were performed in triplicate by mixing 1 μL of RT product with 10 μL of SYBR qPCR Mastermix (Qiagen, Hilden, Germany) containing the appropriate primers (345 nM of a forward primer and 345 nM of a reverse primer). Thermal cycling and fluorescence monitoring were performed in a CFX96 (Bio-rad, Hercules, CA, USA) using the following cycling conditions: 95 °C for 10 min and (95 °C for 15 s, 60 °C for 1 min) × 40. Values were normalized with *Rpl14*. The primers used were as follows:
RpL14F: 5′- TTGCACGTTTCCTCTGTGACG -3′;R5′- TCAGAAGGCTCCACCTCCAA -3′;dSLC5A5F: 5′- TCTCTGCCGCTATTGGAGTT -3′;R5′- TTCCGTAGGCGTACATTTCC -3′

### 4.15. Diet Feeding Regimen

The normal diet (ND) and the high-sugar diet (HSD) were prepared as described in [37]. The feeding regimen using both diets was as follows (see also Figure S7A): Parental flies including w^1118^ flies, RNAi transgene control flies, *Gal4* control flies, or progeny flies from the *Gal4/UAS* crosses were reared on an ND until a single uniform of eggs were laid on the food surface (typically in ~2 days). The parents were then removed, and the progenies continued to be reared on the same ND until eclosion. Newly eclosed male and female adults (equal ratio) were collected and transferred to fresh ND vials or high-sugar-diet (HSD) vials for rearing for 10 days before being processed for the various analyses.

### 4.16. Food Intake Measurement

Food intake for adult flies was measured using the capillary feeder (CAFE) assay as described previously [38]. Briefly, 1-week-old adult female flies (*n* = 10) were weighed and then starved for 16 h before being transferred to the CAFE chambers comprising an empty food vial with wet cotton at the bottom to maintain humidity. One 100 µL (1 µL/mm) capillary tube with liquid food (400 mM sucrose solution + blue dye) was inserted into each chamber through a cotton plug. Flies were fed at room temperature for 6 h, after which total liquid food consumption was calculated as the length of colored food at 0 h minus the length of colored food at 6 h within the capillary tube (ΔL). A control liquid food chamber without flies was used for the correction of liquid food evaporation (ΔL′). Total food consumption (ΔL–ΔL′) in μL was normalized to weight (g). Food consumption per fly was calculated by further dividing the normalized total food consumption by 10.

### 4.17. Structural Analyses

Sequence alignment and analysis of protein primary structures, including prediction of transmembrane helix, putative N-glycosylation sites, and Na-binding sites, were performed by UniProt. Available online: https://www.uniprot.org/(accessed on 10 August 2019), InterPro. Available online: https://www.ebi.ac.uk/interpro/(accessed on 11 August 2019), and ExPASy. Available online: https://www.expasy.org/(accessed on 19 September 2019) software with protein sequences in FASTA format extracted from National Center for Biotechnology Information (NCBI). The analyzing tool Protter. Available online: http://wlab.ethz.ch/protter/start/(accessed on 22 June 2019) is used to predict secondary structures of proteins, in which only a custom protein sequence in FASTA format or the UniProt protein accession of the protein is needed for the analysis [39].

### 4.18. Statistical Analysis

All data were presented as the mean ± SEM of the indicated number of replicates. Pairwise comparison *p*-values involving only 2 groups (control and experimental groups) were analyzed using unpaired, two-tailed, Student’s *t*-test. Statistical analysis was performed using Microsoft Excel (2016). *p* < 0.05 was considered statistically significant.

## 5. Conclusions

Although four genes of the dSLC5A5 family were identified as putative orthologs of human *SLC5A1* and share the highest homology to hSGLT1, this study has only investigated dSLC5A5, one of the four genes. The roles of the other three genes in intestinal glucose uptake and glucose homeostasis maintenance remain to be studied in future work.

## Supplementary Materials

The following are available online at https://www.mdpi.com/article/10.3390/ijms222212424/s1.
