# Supplementary material for: Drosophila Solute Carrier 5A5 Regulates Systemic Glucose Homeostasis by Mediating Glucose Absorption in the Midgut
Source: Int J Mol Sci. 2021 Nov 17;22(22):12424. doi: 10.3390/ijms222212424 (PMC8617630; doi:10.3390/ijms222212424)
Supplement: Supplementary file 1 [file ijms-22-12424-s001.zip › ijms-1436594-sm.pdf]

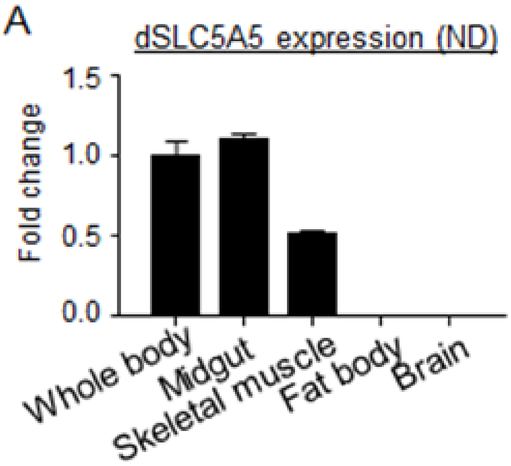

Figure S1. Tissue expression patterns of *dSLC5A5* on normal and high sugar diets. (A) Quantitative real-time PCR analysis of *dSLC5A5* levels in different tissues in 1-week-old female *wild-type* (*w*^{1118}) flies reared on normal diet (ND). *RPL14* served as an internal control. Results are the mean ± SEM of 15 flies analyzed over 3 independent experiments and expressed as the fold change compared with that of whole-body *dSLC5A5* mRNA level (set at 1.0).

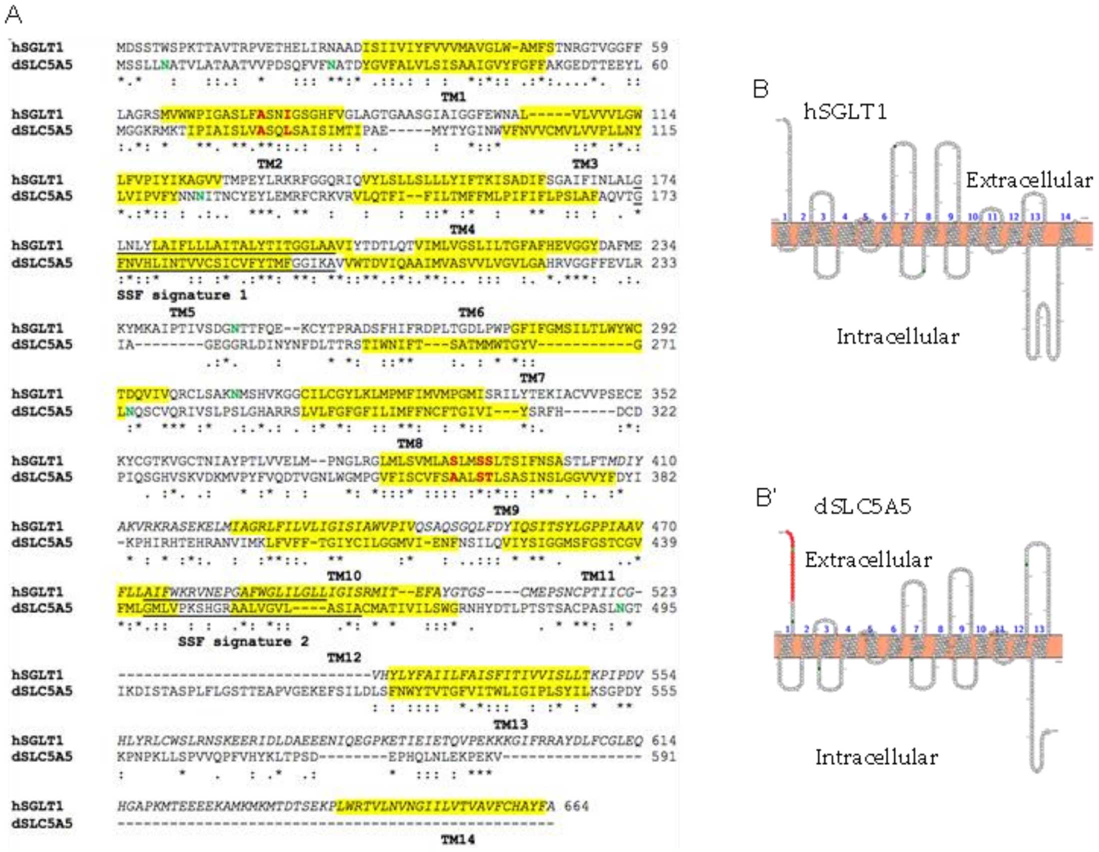

Figure S2. Similarities between hSGLT1 and dSLC5A5. (A) Alignment of amino acid sequences of hSGLT1 and dSLC5A5 produced by UniProt, InterPro and ExPASy. Fully conserved amino acids residues are shown in (*), strongly conserved residues are shown in (:) and weakly conserved residues are shown in (.). Transmembrane regions are shaded in yellow. Sodium: solute symporter family (SSF) signatures are underlined. Putative N-glycosylation sites are in green. Na-binding sites are in

red. (B-B′) The secondary structures of hSGLT1 (B) and dSLC5A5 (B′). The signal peptide is highlighted in red. Putative N-glycosylation sites are in green.

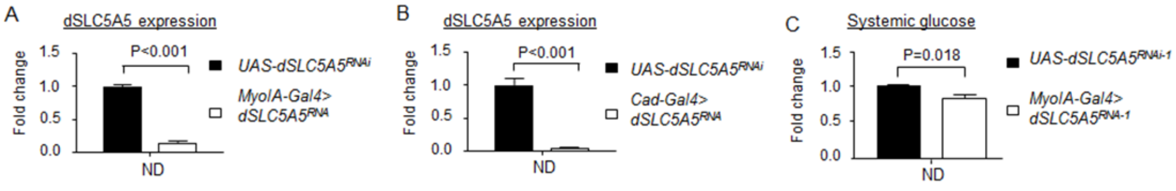

Figure S3. Extent of *dSLC5A5* knockdown mediated by different *Gal4* drivers. (A, B) Quantitative real-time PCR analysis of *dSLC5A5* levels in the midgut of RNAi transgene control flies (*UAS-dSLC5A5^{RNAi}*) or in midgut-specific *dSLC5A5*-KD flies induced by *MyoIA-Gal4* (A) or *Caudal-Gal4* (B) on normal diet (ND). *RPL14* served as an internal control. Results are the mean ± SEM of midguts isolated from 30-40 flies analyzed over 3 independent experiments and expressed as the fold change compared with that on ND (set at 1.0). Student's *t*-test was used to derive *P*-values between the control and *Gal4*-mediated KD flies. (C) Systemic glucose levels in control flies or in midgut-specific *dSLC5A5*-KD flies mediated by *MyoIA-Gal4*. Results are the mean ± SEM of 30-40 flies analyzed over at least 5 independent experiments and expressed as the fold change compared with that of the control flies (set at 1.0). Student's *t*-test was used to derive *P*-values between the transgene control and KD flies.

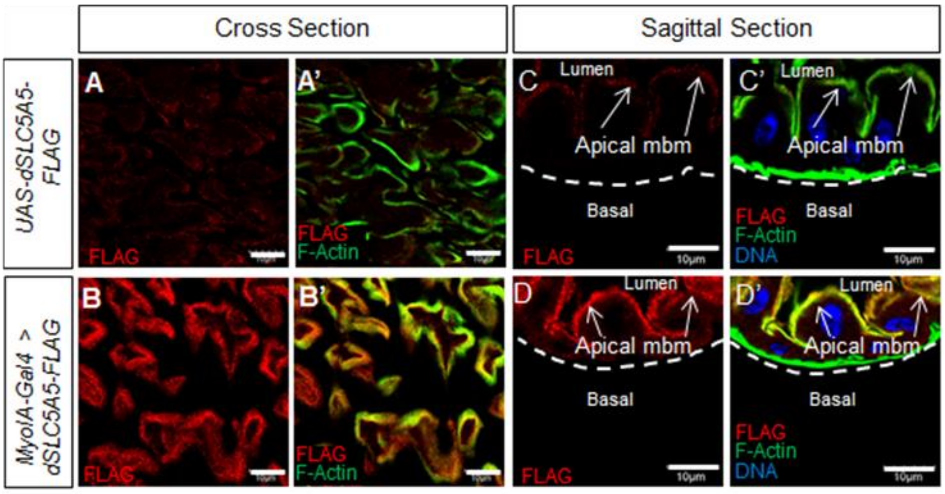

Figure S4. Increased apical membrane abundance of dSLC5A5 in dSLC5A5-overexpressing midgut enterocytes. (A-D′) Representative confocal images of the cross sections (A-B′) or sagittal sections (C-D′) of midguts (R4c-R5 region) in the transgene control flies (*UAS-dSLC5A5-FLAG*) (A-A′) or in midgut-specific *dSLC5A5*-overexpressing flies mediated by *MyoIA-Gal4* (B-B′). In all cases, midguts were dissected from 1-week old flies and immunostained for FLAG (red), F-actin (green) and DNA (blue). Scale bars represent 10 μm.

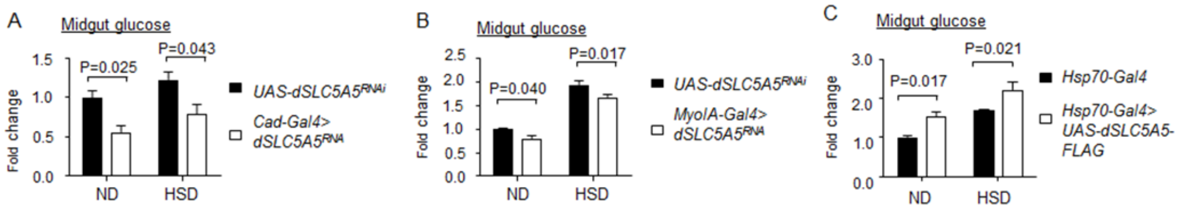

Figure S5. Midgut-specific knockdown or overexpression of dSLC5A5 decreases or increases, respectively, midgut glucose levels. (A-B) Midgut glucose levels in RNAi transgene control flies (*UAS-dSLC5A5^{RNAi}*) or in midgut-specific *dSLC5A5*-KD flies induced by *Caudal-Gal4* (*Cad-Gal4>dSLC5A5^{RNAi}*) (A) or *MyoIA-Gal4* (*MyoIA-Gal4>dSLC5A5^{RNAi}*) (B) on normal diet (ND) or high

sugar diet (HSD). (C) Midgut glucose levels in 1-week old *Gal4* driver control flies (*Hsp70-Gal4*) or in whole body-*dSLC5A5-FLAG* overexpressing flies mediated by *Hsp70-Gal4* (*Hsp70-Gal4>dSLC5A5-FLAG*) on ND or HSD. In all cases, midgut glucose levels (µg/µl) were normalized to midgut protein levels (µg/µl). Results are expressed as the mean ± SEM from at least five biological replicates, with 10-12 midguts analyzed for each biological replicate. Student's *t*-test was used for statistical analysis between control and KD- or overexpressing flies under ND or HSD condition.

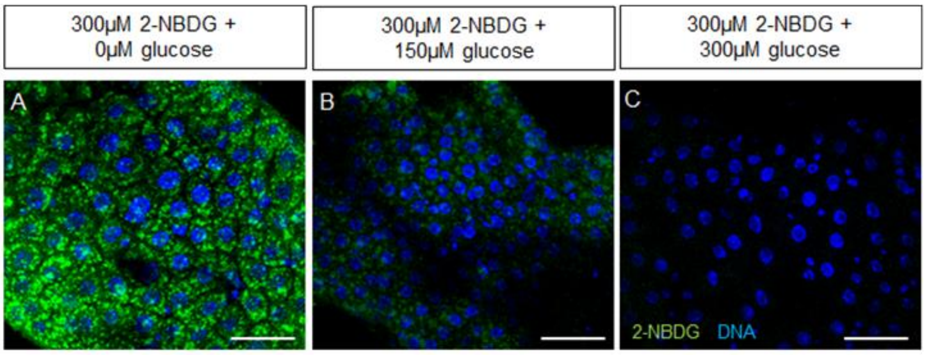

Figure S6. 2-NBDG and glucose are absorbed through the glucose transporter in midgut enterocytes. (A-C) Representative confocal images of midguts (R4c-R5 region) dissected from female w^{1118} flies and incubated with 300 µM 2-NBDG together with 0 µM (A), 150 µM (B), and 300 µM (C) of glucose for 30 minutes at room temperature following a 30-minute starvation in PBS. Scale bars represent 30 µm.

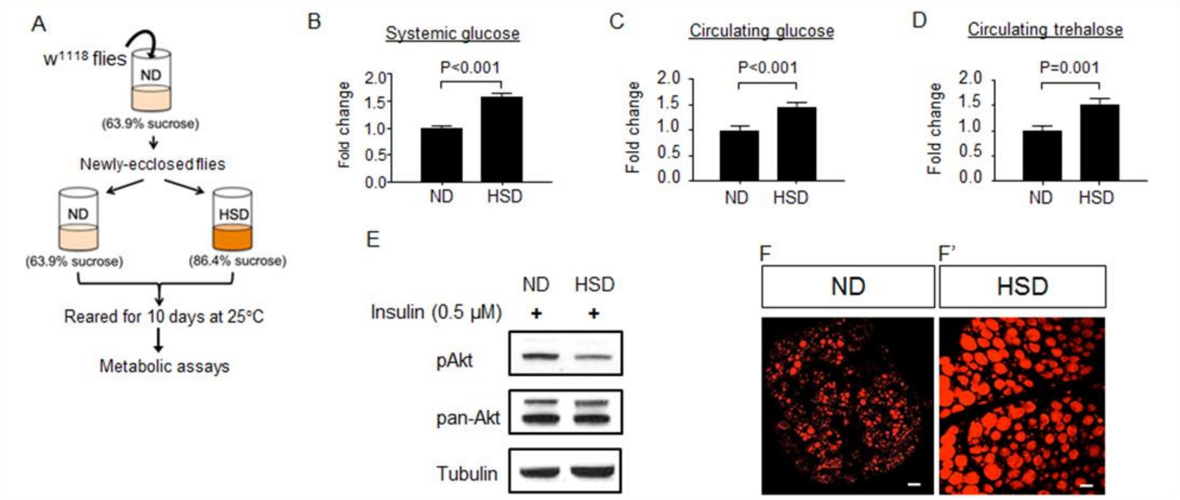

Figure S7. High sugar diet (HSD) feeding of *Drosophila* generates metabolic disorders. (A) Schematic workflow of the high sugar diet (HSD) feeding regimen of wild-type w^{1118} flies. (B-D) Levels of whole-body glucose (B), circulating glucose (C), and circulating trehalose (D) in w^{1118} flies after 10-day feeding on normal diet (ND) or HSD post-eclosion. Systemic glucose levels (µg/µl) were normalized to total protein of whole flies (µg/µl). Results are the mean ± SEM of 30-40 flies analyzed over at least 5 independent experiments and are expressed as the fold change compared with that of the control flies (set at 1.0). Student's *t*-test was used for *P*-value analysis. (E) Western blot analysis of phosphorylated Akt (p-Akt) level in 1-week old w^{1118} flies on ND or HSD after 15-minute incubation with 0.5 µM human insulin. (F-F') Representative confocal images of lipid droplets stained by Nile Red in the fat body of w^{1118} flies under ND (F) or HSD condition (F'). Scale bars represent 20 µm.

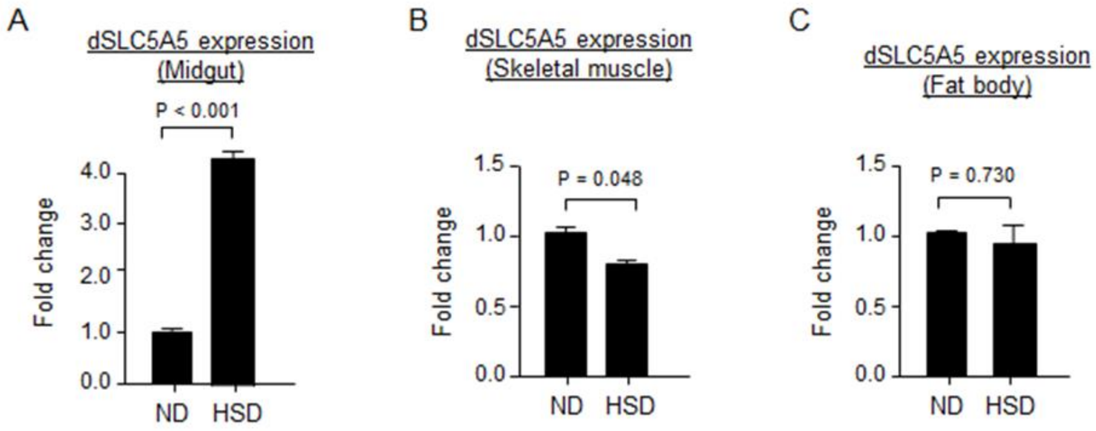

Figure S8. Midgut DSLC5A5 transcript levels under normal diet (ND) and high sugar diet (HSD). (A-C) Quantitative real-time PCR analysis of *dSLC5A5* levels in the midgut (A), skeletal muscle (B), or fat body (C) in 1-week-old female *wild-type* (w^{1118}) flies reared on normal diet (ND) and high sugar diet (HSD). *RPL14* served as an internal control. Results are the mean ± SEM of tissues isolated from 30-40 flies analyzed over 3 independent experiments and expressed as the fold change compared with that on ND (set at 1.0). Student's *t*-test was used to derive *P*-values between normal diet (ND) and high sugar diet (HSD) conditions.

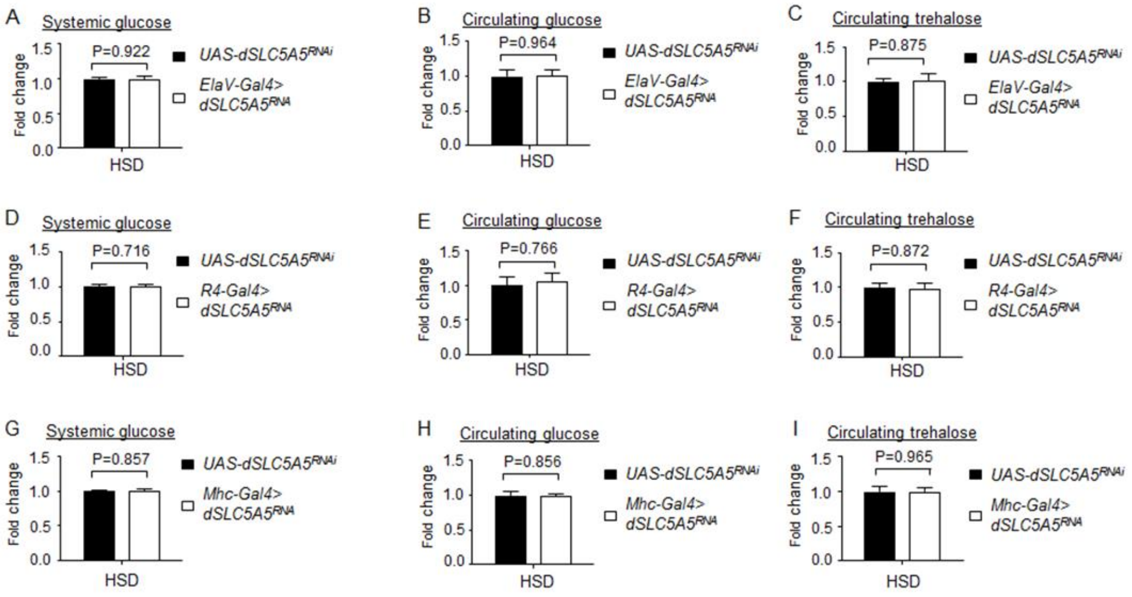

Figure S9. dSLC5A5 inhibition in the brain, fat body, or skeletal muscle does not affect systemic and circulating glucose levels under high sugar diet (HSD) condition. (A, D, G) Systemic glucose levels in RNAi transgene control flies (*UAS-dSLC5A5*^{RNAi}) or flies bearing the neuronal KD of *dSLC5A5* using *ElaV-Gal4* (A), the fat body KD of *dSLC5A5* using *R4-Gal4* (D), or the skeletal muscle KD of *dSLC5A5* using *Mhc-Gal4* (G) on HSD. (B, E, H) Circulating glucose levels in control flies (*UAS-dSLC5A5*^{RNAi}), flies bearing the neuronal KD of *dSLC5A5* using *ElaV-Gal4* (B), flies bearing the fat body KD of *dSLC5A5* using *R4-Gal4* (E), or flies bearing the skeletal muscle KD of *dSLC5A5* using *Mhc-Gal4* (H), on HSD. (C, F, I) Circulating trehalose levels in control flies (*UAS-dSLC5A5*^{RNAi}) or flies bearing the neuronal KD of *dSLC5A5* using *ElaV-Gal4* (C), flies bearing the fat body KD of *dSLC5A5* using *R4-Gal4* (F), or flies bearing the skeletal muscle KD of *dSLC5A5* using *Mhc-Gal4* (I), on HSD. In A, D, and G, systemic glucose levels (μg/μl) were normalized to whole-body protein (μg/μl). Results are the mean ± SEM of 30-40 flies analyzed over at least 5 independent experiments and expressed as the fold change compared with that of the control flies (set at 1.0). Student's *t*-test was used to derive *P*-values between the transgene control and KD flies on HSD.
