# Supplementary figures and images for: Drosophila Solute Carrier 5A5 Regulates Systemic Glucose Homeostasis by Mediating Glucose Absorption in the Midgut
Source: Int J Mol Sci. 2021 Nov 17;22(22):12424. doi: 10.3390/ijms222212424 (PMC8617630; doi:10.3390/ijms222212424)

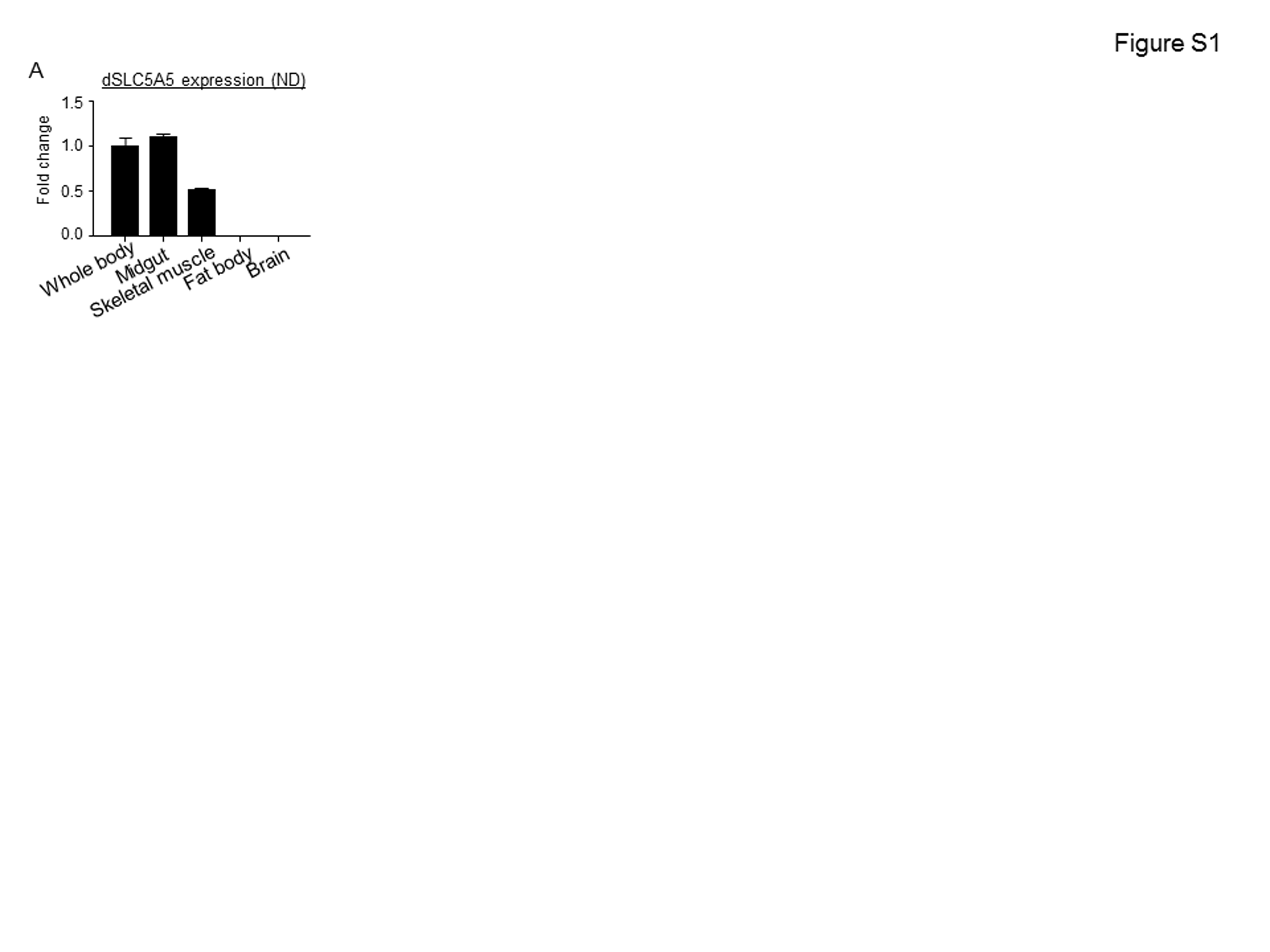

Supplement: Supplementary file 1 [file ijms-22-12424-s001.zip › Slide1.TIF]

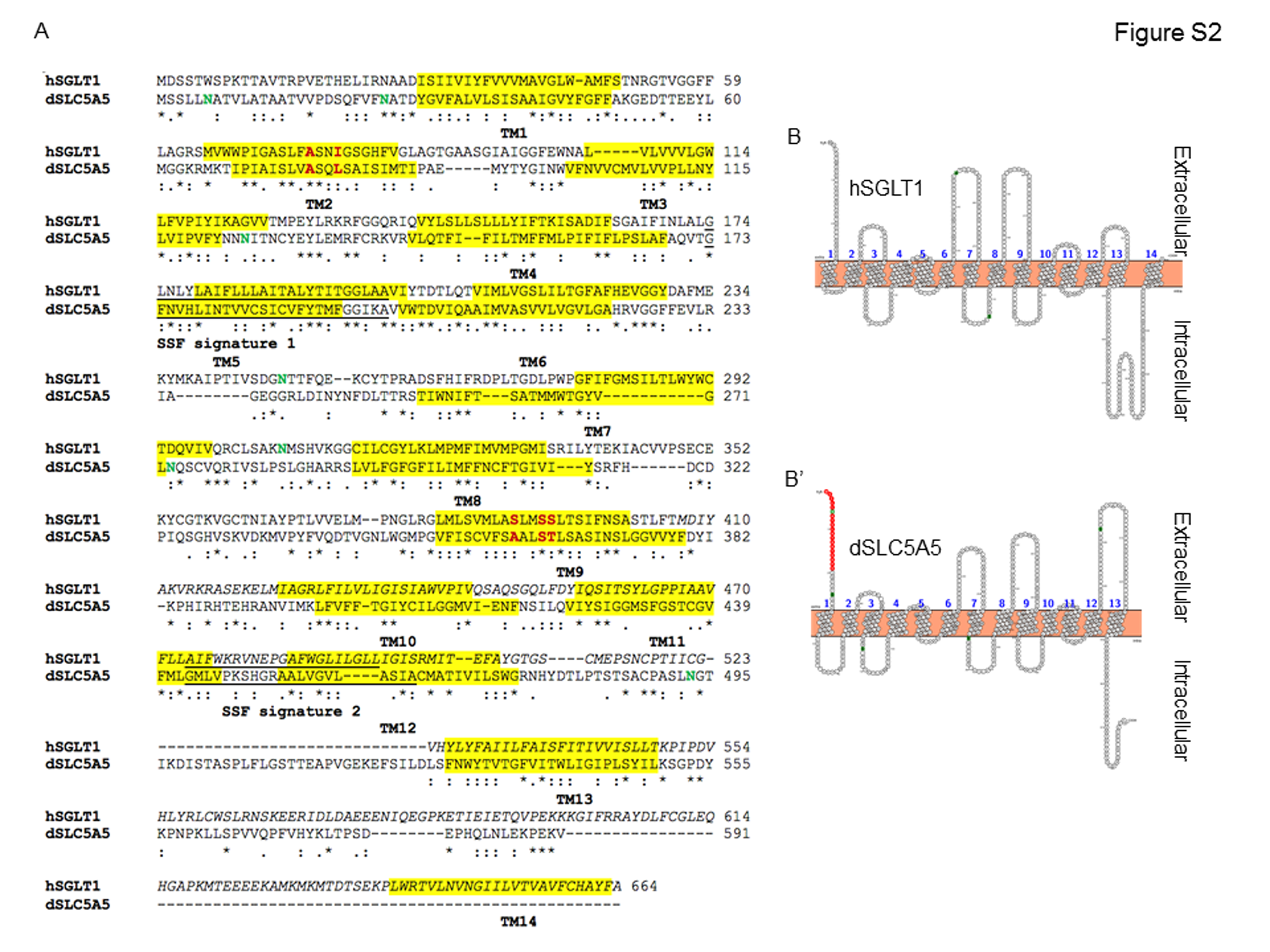

Supplement: Supplementary file 1 [file ijms-22-12424-s001.zip › Slide2.TIF]

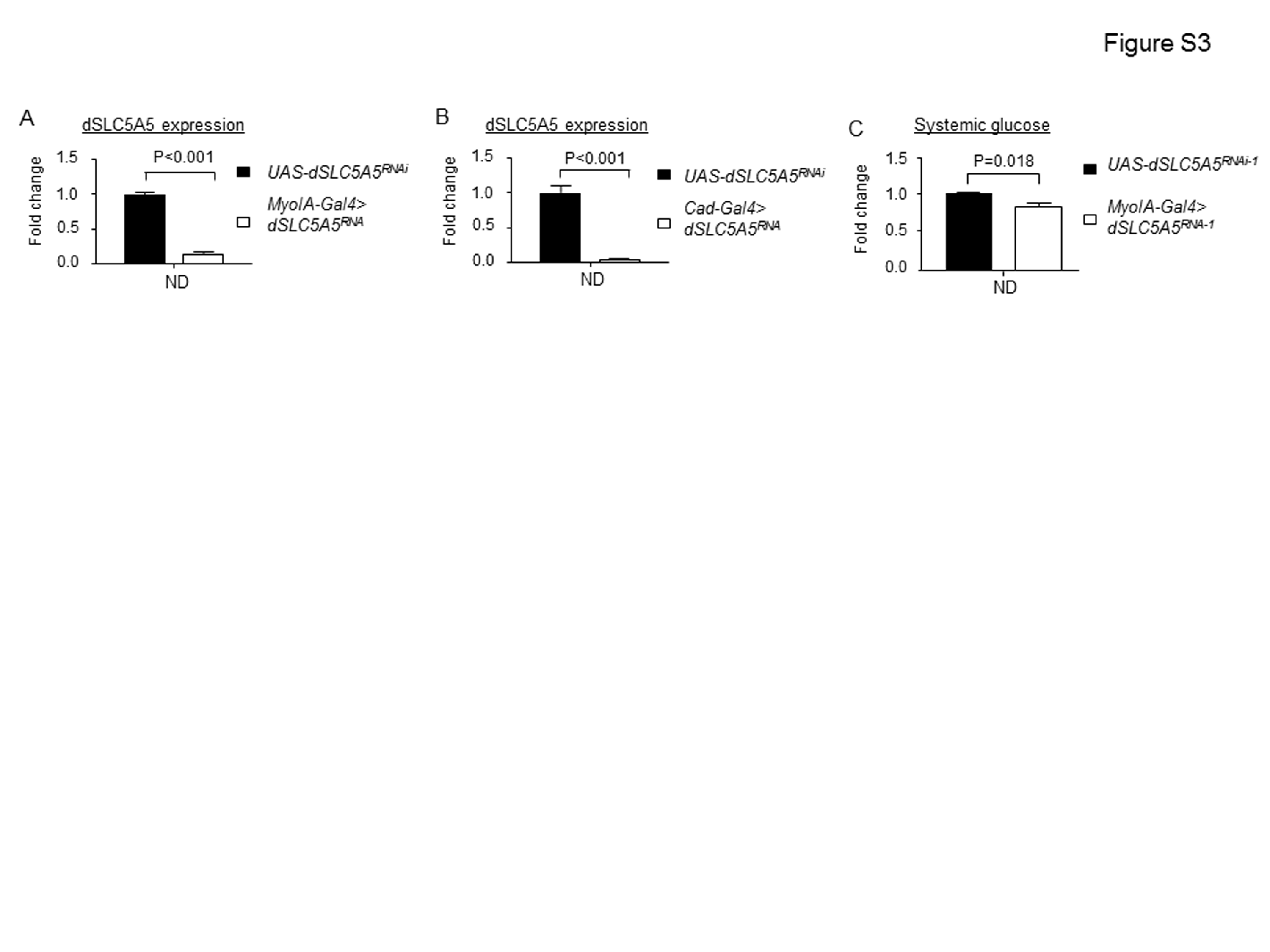

Supplement: Supplementary file 1 [file ijms-22-12424-s001.zip › Slide3.TIF]

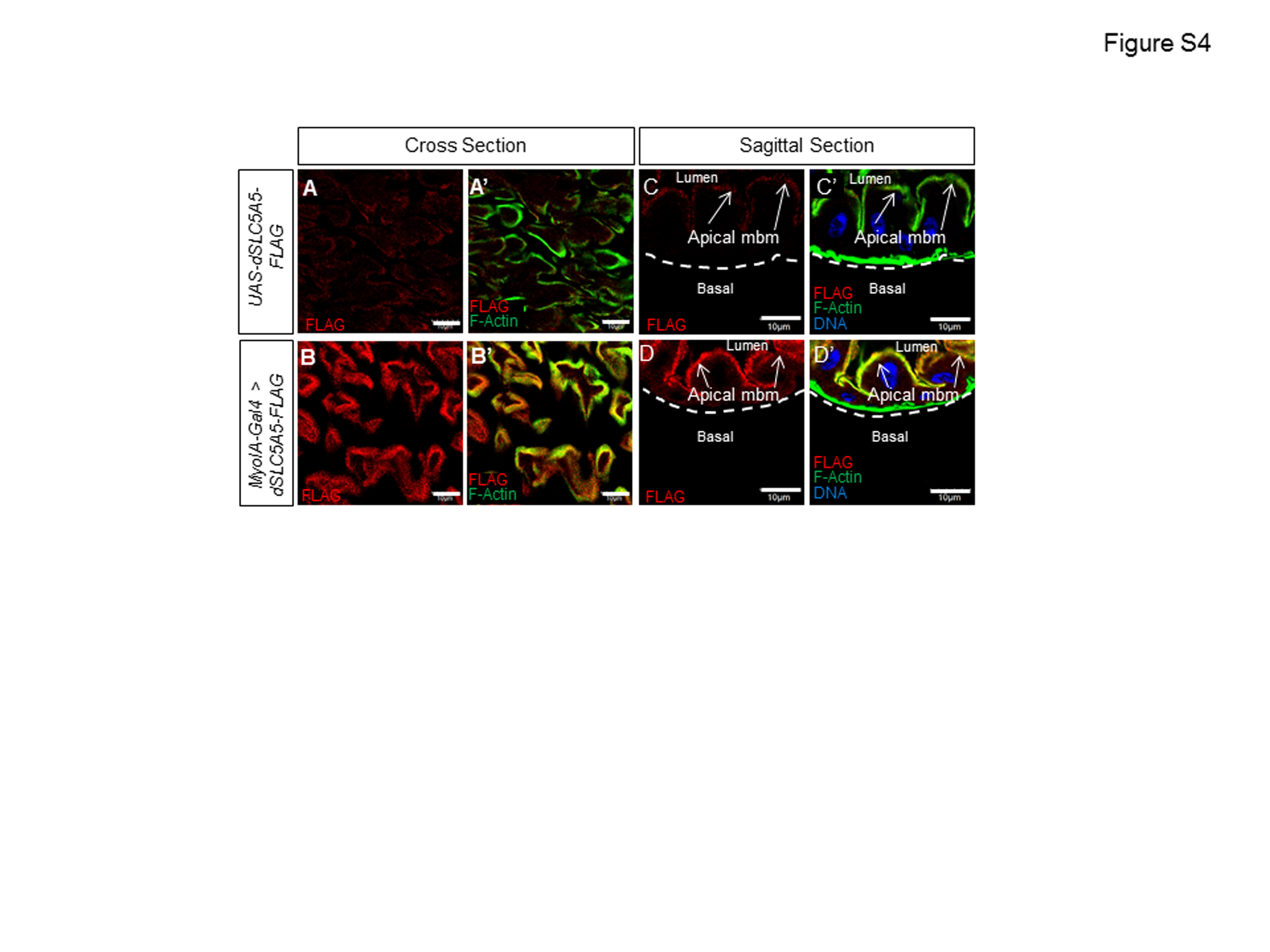

Supplement: Supplementary file 1 [file ijms-22-12424-s001.zip › Slide4.TIF]

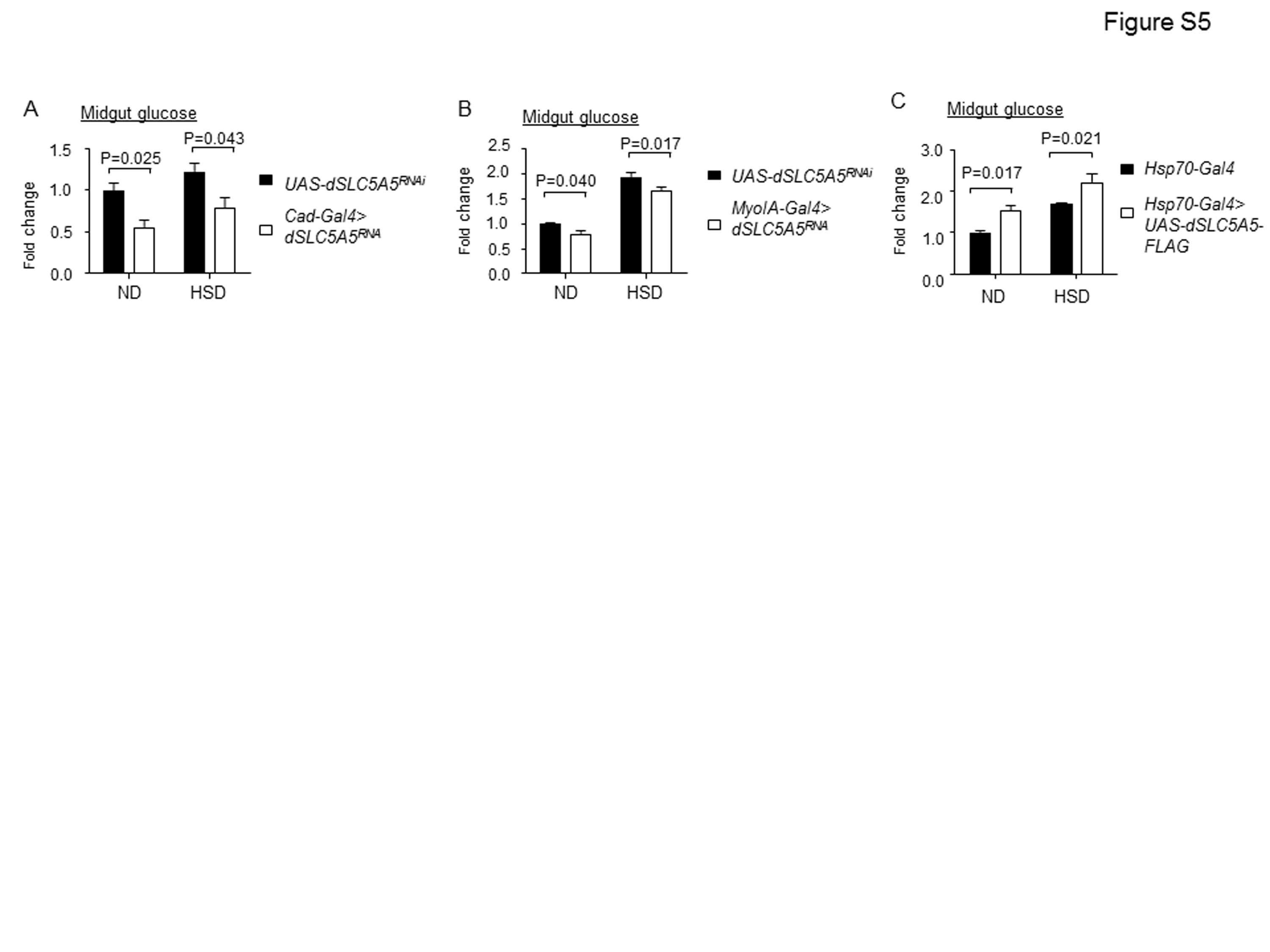

Supplement: Supplementary file 1 [file ijms-22-12424-s001.zip › Slide5.TIF]

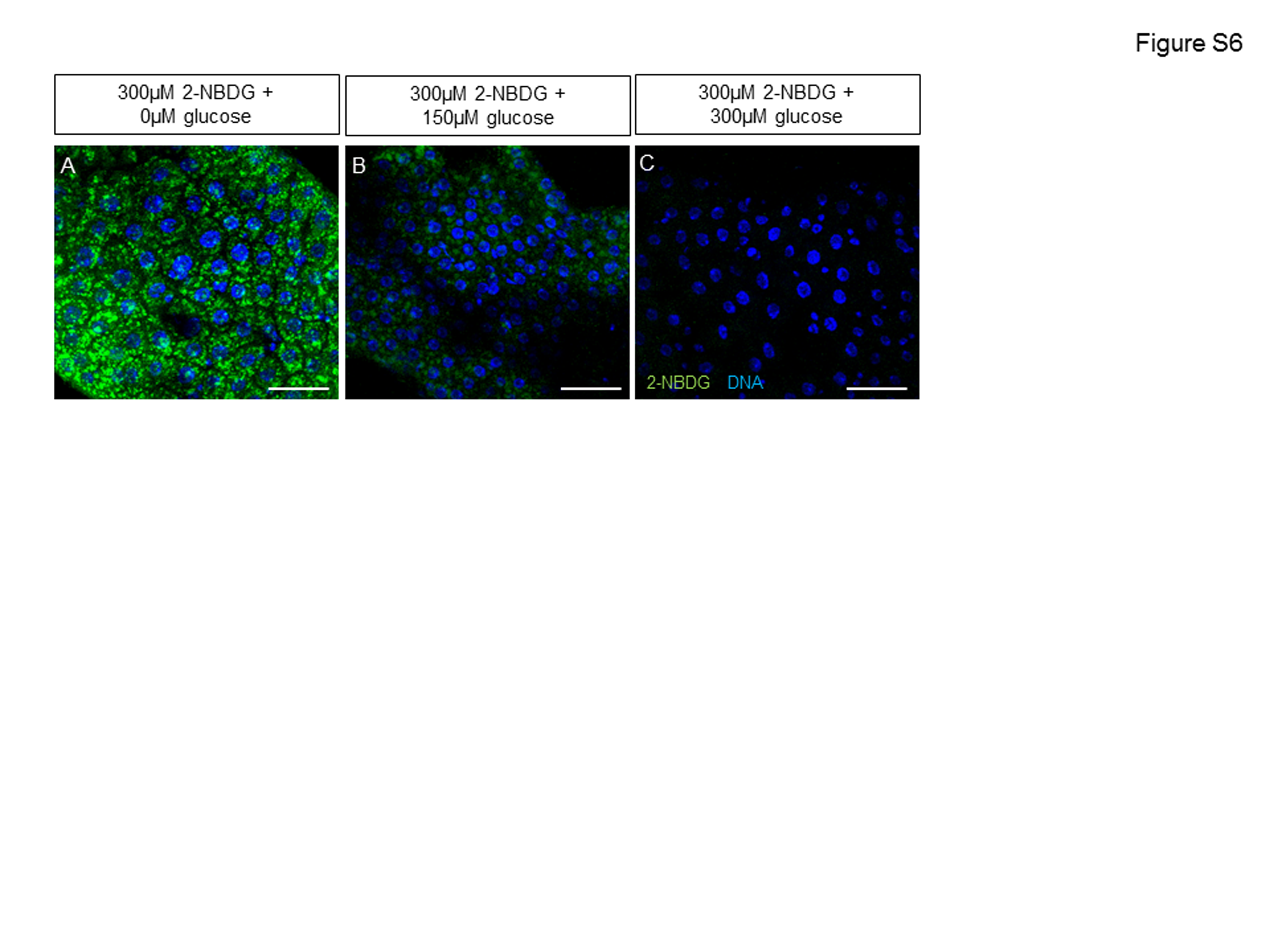

Supplement: Supplementary file 1 [file ijms-22-12424-s001.zip › Slide6.TIF]

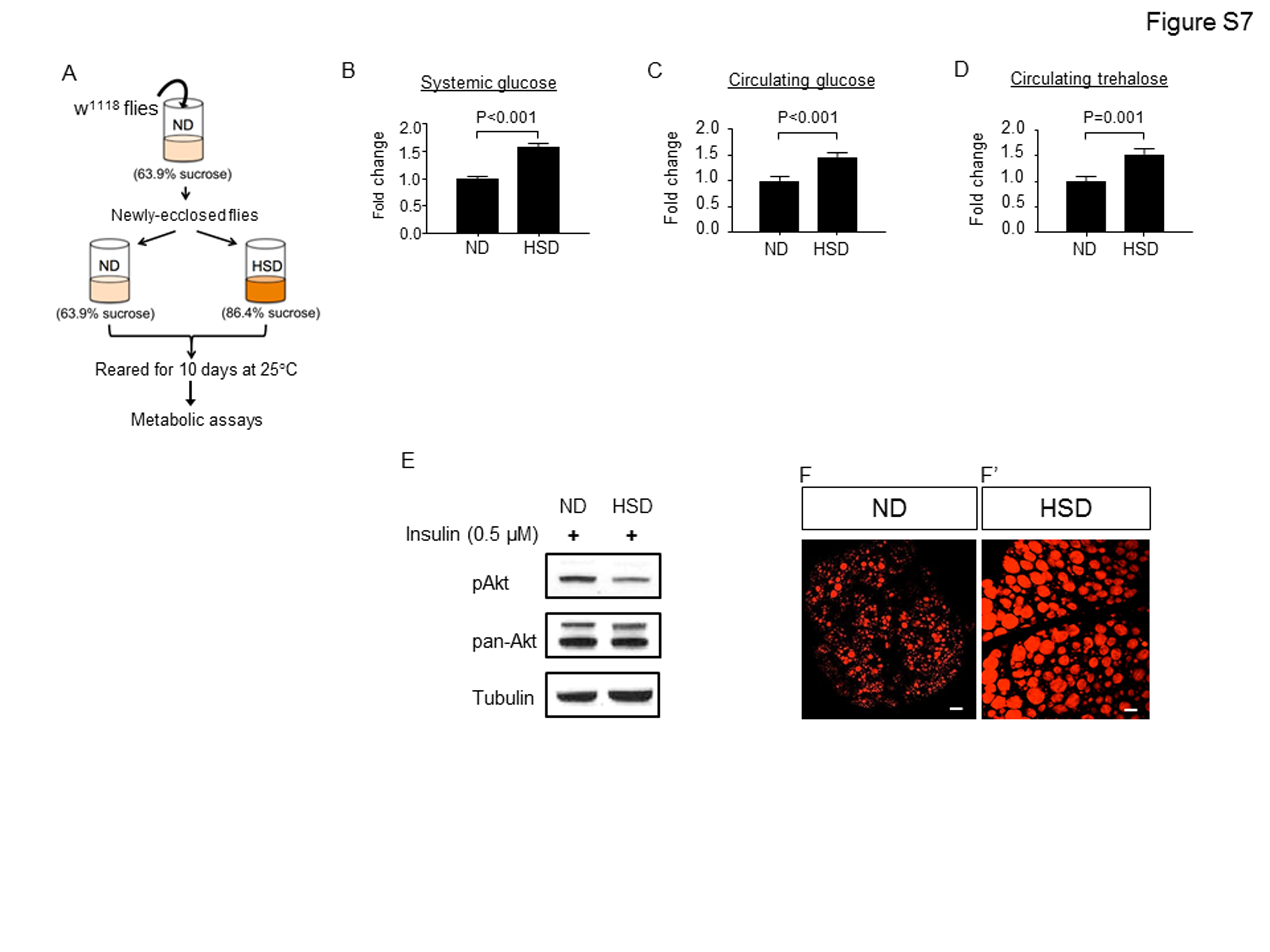

Supplement: Supplementary file 1 [file ijms-22-12424-s001.zip › Slide7.TIF]

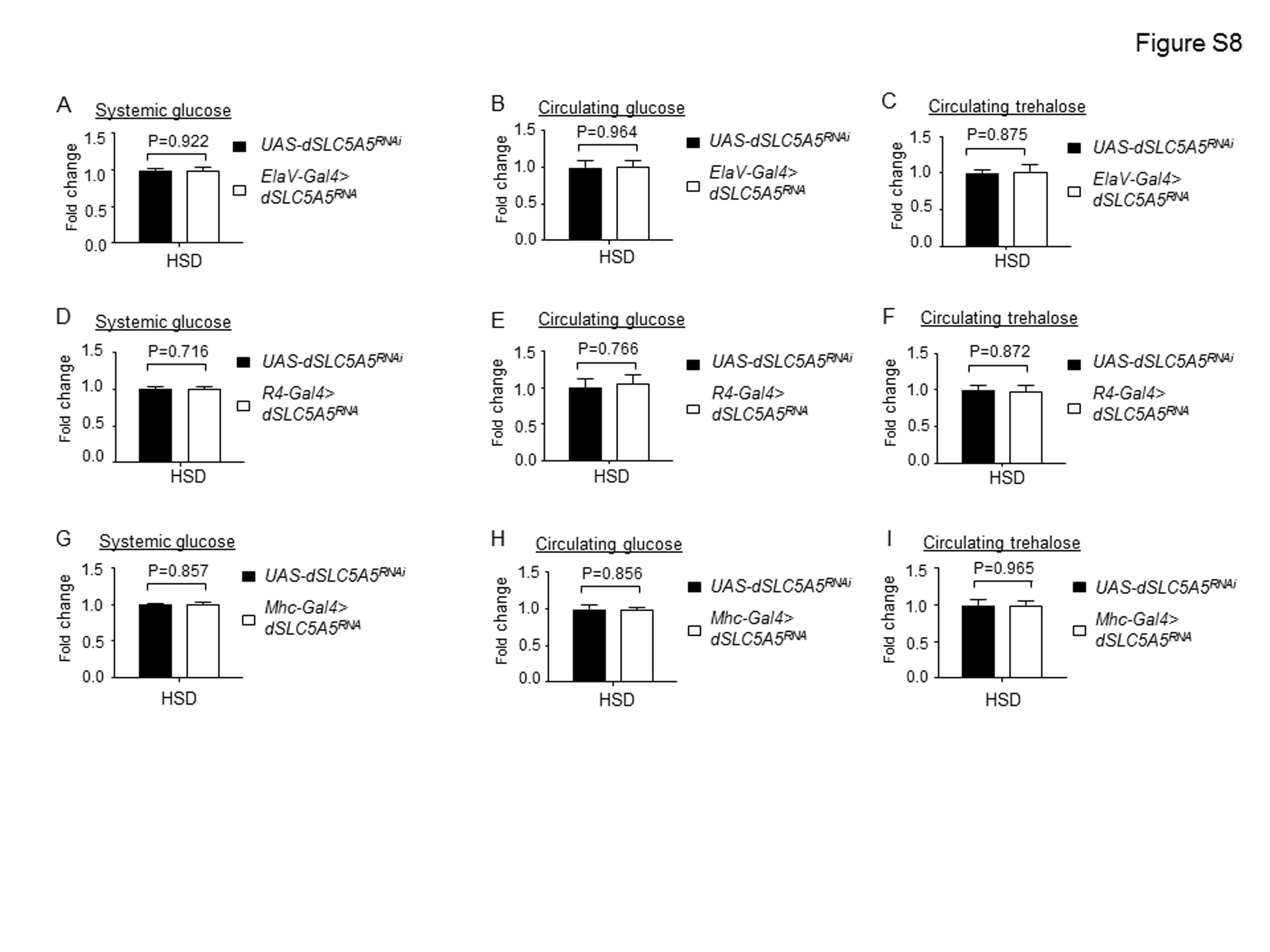

Supplement: Supplementary file 1 [file ijms-22-12424-s001.zip › Slide8.TIF]

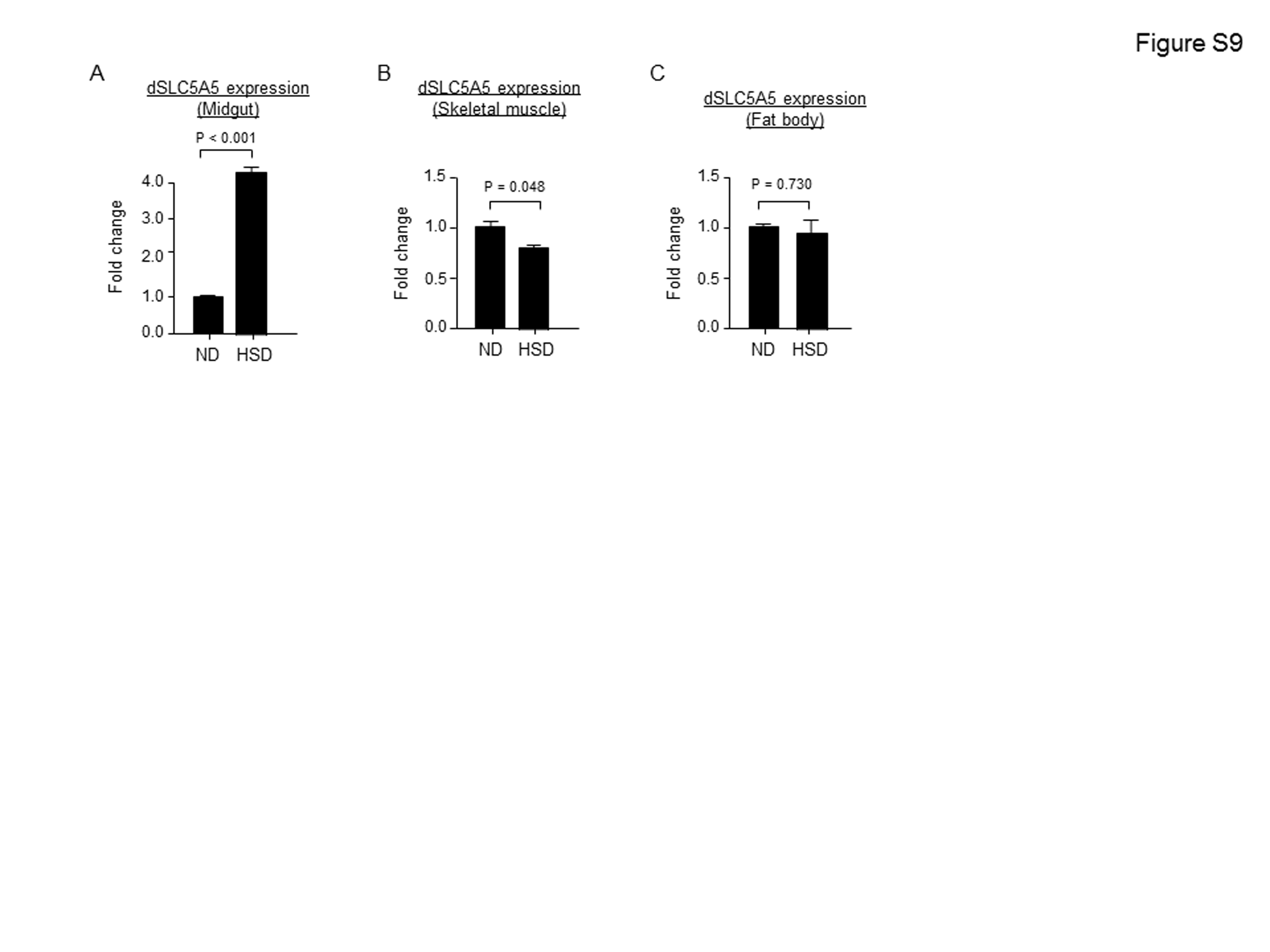

Supplement: Supplementary file 1 [file ijms-22-12424-s001.zip › Slide9.TIF]
